# De novo transcriptome assembly from the gonads of a scleractinian coral, *Euphyllia ancora*: molecular mechanisms underlying scleractinian gametogenesis

**DOI:** 10.1186/s12864-020-07113-9

**Published:** 2020-10-21

**Authors:** Yi-Ling Chiu, Shinya Shikina, Yuki Yoshioka, Chuya Shinzato, Ching-Fong Chang

**Affiliations:** 1 Doctoral Program in Marine Biotechnology, National Taiwan Ocean University, Keelung, 20224 Taiwan; 2 Doctoral Program in Marine Biotechnology, Academia Sinica, Taipei, 11529 Taiwan; 3 Institute of Marine Environment and Ecology, National Taiwan Ocean University, Keelung, Taiwan; 4 Center of Excellence for the Oceans, National Taiwan Ocean University, 2 Pei-Ning Rd, Keelung, 20224 Taiwan; 5 Atmosphere and Ocean Research Institute, The University of Tokyo, Chiba, 277-8564 Japan; 6 Department of Aquaculture, National Taiwan Ocean University, Keelung, Taiwan

**Keywords:** Scleractinian corals, *Euphyllia ancora*, Ovary, Testis, Gonads, RNA-seq, Transcriptome assembly, Sex-specific, Phase-specific, Oogenesis, Spermatogenesis

## Abstract

**Background:**

Sexual reproduction of scleractinians has captured the attention of researchers and the general public for decades. Although extensive ecological data has been acquired, underlying molecular and cellular mechanisms remain largely unknown. In this study, to better understand mechanisms underlying gametogenesis, we isolated ovaries and testes at different developmental phases from a gonochoric coral, *Euphyllia ancora*, and adopted a transcriptomic approach to reveal sex- and phase-specific gene expression profiles. In particular, we explored genes associated with oocyte development and maturation, spermiogenesis, sperm motility / capacitation, and fertilization.

**Results:**

1.6 billion raw reads were obtained from 24 gonadal samples. De novo assembly of trimmed reads, and elimination of contigs derived from symbiotic dinoflagellates (Symbiodiniaceae) and other organisms yielded a reference *E. ancora* gonadal transcriptome of 35,802 contigs. Analysis of 4 developmental phases identified 2023 genes that were differentially expressed during oogenesis and 678 during spermatogenesis. In premature/mature ovaries, 631 genes were specifically upregulated, with 538 in mature testes. Upregulated genes included those involved in gametogenesis, gamete maturation, sperm motility / capacitation, and fertilization in other metazoans, including humans. Meanwhile, a large number of genes without homology to sequences in the SWISS-PROT database were also observed among upregulated genes in premature / mature ovaries and mature testes.

**Conclusions:**

Our findings show that scleractinian gametogenesis shares many molecular characteristics with that of other metazoans, but it also possesses unique characteristics developed during cnidarian and/or scleractinian evolution. To the best of our knowledge, this study is the first to create a gonadal transcriptome assembly from any scleractinian. This study and associated datasets provide a foundation for future studies regarding gametogenesis and differences between male and female colonies from molecular and cellular perspectives. Furthermore, our transcriptome assembly will be a useful reference for future development of sex-specific and/or stage-specific germ cell markers that can be used in coral aquaculture and ecological studies.

## Background

Since the discovery of scleractinian mass spawning events in the Great Barrier Reef in the 1980s [1–3], sexual reproduction of scleractinians has captured the attention of researchers and the general public. Studies on various aspects of sexual reproduction, such as the timing of broadcast spawning or brooding, general cellular processes of gametogenesis, and sexuality (hermaphroditic or gonochoric), have been undertaken mainly from an ecological perspective in many scleractinian species in many locations during the past 3 decades [4–6]. Although large amounts of data are now available from more than four hundred species [7], our current understanding of intrinsic mechanisms underlying key processes of sexual reproduction, such as sex determination/differentiation, gametogenesis, and ovulation/spawning, is quite limited.

Gametogenesis is a highly organized process whereby genetically diverse haploid gametes are created from diploid germ cells through meiosis with recombination. Generally, scleractinian germ cells are developed in endodermal mesenteries of polyps [4, 8, 9]. Sites of germ cell development are often observed as swellings in polyps during active gametogenesis, and are termed gonads [4]. Oogenesis begins with mitotic division of a small number of oogonia along the gonadal mesoglea, a thin layer composed of extracellular matrix. After oogonia differentiate into oocytes by entering a meiotic phase, oocytes increase in size and migrate into the mesoglea layer [4, 9–11]. There, oocytes further increase in size until maturation by accumulating yolk proteins, lipids, and other essential materials for embryonic development [12, 13]. Spermatogenesis begins with the active mitotic division of spermatogonia in the gonadal mesogleal layer. After spermatogonia form many small clusters comprising dozens of spermatogonia, they migrate into the mesoglea layer and form many spermatogenic compartments called spermaries. Further proliferation of spermatogonia, meiotic differentiation into spermatocytes, and spermiogenesis take place within each spermary [4, 14].

Studies of molecular and cellular aspects of scleractinian gametogenesis have just recently begun. Only several reports are available describing genes related to oogenesis, including vitellogenesis [12–20] and spermatogenesis [21, 22]. Currently, in order to cope with recent declines of coral reefs, reef restoration efforts via aquaculture are being initiated worldwide [23–25]. A comprehensive understanding of intrinsic mechanisms of gametogenesis will enable us to approach coral reef restoration from a new perspective. For instance, hormonal induction of gametogenesis and spawning under artificial rearing systems would allow more efficient propagation of target species [14]. Sex-and stage-specific molecular markers for germ cells would also enable us to monitor and to evaluate the developmental status of germ cells in corals cultured in captivity [21]. Moreover, because scleractinians belong to the phylum Cnidaria (e.g., corals, sea anemones, hydras, and jellyfish), which are regarded as evolutionarily basal in the animal kingdom, studies highlighting common mechanisms of sexual reproduction between scleractinians and advanced animals (e.g., vertebrates) should provide insights into the evolution of sexual reproduction in metazoans [14].

**Fig. 1:**
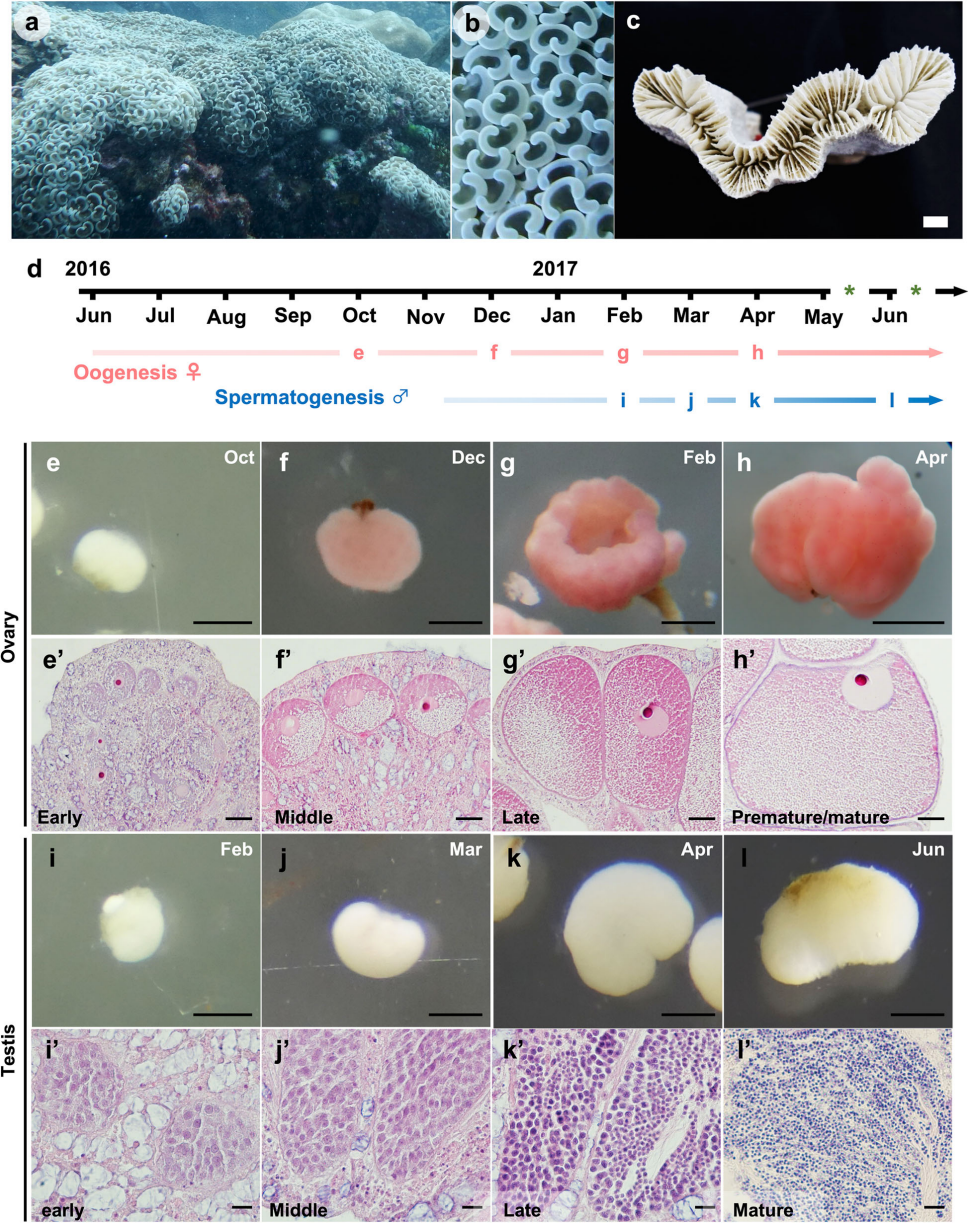
*Euphyllia ancora* and its germ cells observed histologically in isolated gonads at different sampling times. **a** External appearance of an *E. ancora* colony. **b** External appearance of tentacles of an *E. ancora* colony. Anchor-like tentacles and the flabello-meandroid skeleton typify *E. ancora*. The pictures were taken at Nanwan Bay, Kenting National Park, in southern Taiwan in October 2016. **c** A top view of an *E. ancora* skeleton. The picture was taken after removal of polyp tissue in the laboratory. **d** Periods of oogenesis (pink arrow) and spermatogenesis (blue arrow) and predicated spawning timing (*). Letters (**e**-**l**) on the arrows correspond to Figure 1 (**e-l)** below, and indicate the timing (month) of sampling for ovaries and testes. **e-h** The external appearance of isolated ovaries in October and December 2016 and February and April 2017. **e’-h’** Histological observation of the isolated ovaries. **e, e’** The early phase of ovaries. **f, f’** The middle phase of an ovary. **g, g’** The late phase of an ovary. **h, h’** The premature/mature phase of an ovary. **i-l** The external appearance of isolated testes in February, March, April, and June 2017. **i’-l’** Histological observation of isolated testes. **i, i’** The early phase of a testis with spermatogonia. **j, j’** The middle phase of a testis having spermatogonia and primary spermatocytes. **k, k’** The late phase of a testis with secondary spermatocytes and spermatids. **l, l’** The mature phase of a testis with mature sperm. Sections were stained with hematoxylin and eosin. Scale bars = 1 cm (**c**); 500 μm (**e-l**); 50 μm (**e’-h’**); 10 μm (**i’-l’**)

Transcriptome analysis using high-throughput sequencing has greatly enhanced identification of transcripts involved in sexual reproduction in various taxa [26–30]. This study performed gonadal transcriptome sequencing of a scleractinian coral, *Euphyllia ancora*, commonly known as the anchor or hammer coral (Fig. 1 a-c). *E. ancora* was selected for the following reasons: (i) These corals are common in the Indo-Pacific region. (ii) They are gonochoric, and their annual gametogenic cycle in reefs along southern Taiwan has been studied histologically in both male and female colonies [8, 9]. For instance, a single oogenic or spermatogenic cycle in this region takes approximately a year in females and half a year in males. Annual spawning occurs within a week after a full moon in April or May, or occasionally in June. Finally, (iii) They have large polyps (3–5 cm in diameter) that allow us to isolate ovaries and testes with relative ease [12]. This transcriptomic analysis of isolated gonads was undertaken in order to discover genes participating in gametogenesis.

The present study isolated ovaries and testes at different developmental phases from wild *E. ancora* colonies in order to reveal sex- and phase-specific gene expression profiles. In particular, we focused on premature and mature phases of gonads to identify candidate genes associated with oocyte development and maturation, spermiogenesis, sperm motility and capacitation, and fertilization, because of their importance for coral aquaculture (e.g., induction of sexual maturation) and ecological studies (e.g., monitoring germline development or predicting spawning time). These findings may highlight conserved molecular mechanisms of gametogenesis between scleractinians and other animals, including humans.

## Results

### Histological analysis of *E. ancora* gonads collected at different times

Ovaries and testes were isolated from wild colonies at different times during a period of 9 months in 2016–2017 (Fig. 1d). Progress of gametogenesis was histologically confirmed as the spawning season approached (April–June, 2017). Gametogenesis is generally synchronized among polyps in a colony. Histological analysis of isolated ovaries showed that oocytes grew steadily during the 9-month investigation, and that ovaries isolated at 4 sampling dates generally displayed different oocyte developmental stages: October 2016 (oocytes with cytoplasmic polarization, < 125 μm in diameter), December 2016 (oocytes with accumulation of yolk and other components, 126–200 μm in diameter), February 2017 (oocytes with accumulation of yolk and other components, 201–275 μm in diameter), and April 2017 (oocytes with ‘U’-like germinal vesicles or GVBD, > 276 μm in diameter) (Fig. 1d, Table 1). Notably, in the April 2017 samples, most oocyte nuclei had translocated to the peripheral membrane (Fig. 1 e-h), and some oocyte nuclei had disappeared (Additional file 1), indicating that germinal vesicle breakdown (GVBD) had occurred in those oocytes. These ovarian samples were then classified into 4 phases, early, middle, late, and premature/mature, and were used for RNA-seq (Fig. 1 e-h, Table 1).

**Table 1:** Criteria for classification of gonadal phases

Gonad	Phase	The most represented germ cells observed in the gonads	Approximate timings of collection in 2016 to 2017
Ovary	Early	Oocytes with cytoplasmic polarization (<125 μm in diameter)	October, 2016
	Middle	Oocytes with accumulation of yolk and other components (126-200 μm in diameter)	December, 2016
	Late	Oocytes with accumulation of yolk and other components (201-275 μm in diameter)	February, 2017
	Premature/ mature	Oocytes with ‘U’-like germinal vesicles and GVBD (>276 μm in diameter)	April, 2017
Testis	Early	Spermatogonia	February, 2017
	Middle	Spermatogonia and primary spermatocytes	March, 2017
	Late	Secondary spermatocytes and spermatids	April, 2017
	Mature	Sperm	June, 2017

Similarly, testes isolated at the following 4 sampling dates in 2017 possessed germ cells in different developmental stages: February (spermatogonia), March (spermatogonia and primary spermatocytes), April (secondary spermatocytes and spermatids), and June (mature sperm) (Fig. 1 d, Table 1) (Fig. 1 i-l). In the June samples, although a small number of spermaries with both round spermatids and mature sperm were observed in some testes, cytological observation confirmed the presence of morphologically mature sperm (Additional file 1). Testis samples were then classified into 4 phases, early, middle, late, and mature, and were subjected to RNA-seq (Fig. 1 i-l, Table 1).

### De novo transcriptome assembly of *E. ancora* gonads, identification of coral contigs, and functional annotation

1.6 billion raw reads comprising approximately 240 Gb of clean transcriptomic sequencing data were obtained by Illumina paired-end sequencing from the selected 12 testis (3 colonies, 4 time points) and 12 ovary (3 colonies, 4 time points) samples. Clean reads were deposited in the Sequence Read Archive (SRA) of DDBJ under BioProject number PRJDB9831 (Additional file 2). De novo assembly of all clean reads produced 169,272 initial contigs with an average size of 2321 bp and an N50 of 4610 bp. Maximum contig length reached 52,720 bp (Table 2). The assembled transcriptome sequences were also deposited in DDBJ under accession number ICQS01000001-ICQS01169272. In addition, we provided the accession numbers that are included in the reference gonadal transcriptome, *E. ancora* contigs and Symbiodiniaceae as Additional files 3, 4, 5 respectively.

**Table 2:** Summary of transcriptome assemblies in this study

	*E. ancora* holobiont (all contigs)	Assigned as *E. ancora* contigs	Assigned as Symbiodiniaceae contigs	Reference *E. ancora* gonadal transcriptome contigs used for this study
Number of contigs sequences	169,272	95,980 (56.7%)	50,943 (30.1%)	35,802
Total basepair (bp)	392,911,637	328,250,055	53,222,313	125,288,259
Minimum length (bp)	200	200	200	297
Average (bp)	2,321	3,420	1,045	3,500
Maximum unigene length (bp)	52,720	52,720	16,557	43,419
N50 size (bp)	4,610	5,384	1,318	5,019
GC (%)	44.2	41.5	50.6	42.1
BUSCO completeness (%)	99.7	98	65	92.1

**Fig. 2:**
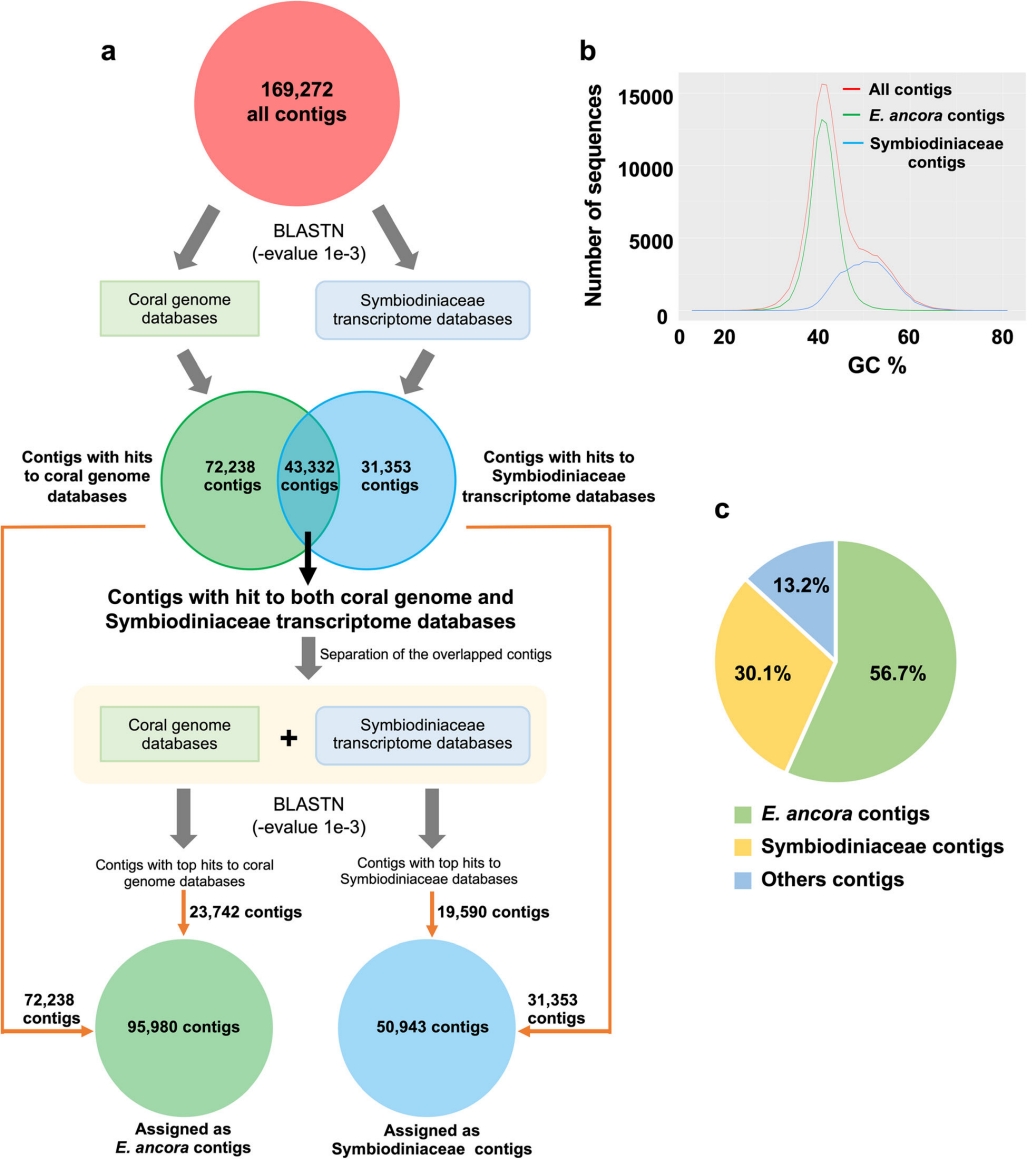
Identification of *E. ancora* contigs from the transcriptome assembly of an *E. ancora* holobiont. **a** A flow chart for identification of *E. ancora* contigs from the transcriptome assembly (all contigs) that contains contigs from the host coral, symbiotic (dinoflagellates), and other organisms (bacteria). **b** Distribution of GC percentages of assembled contigs. Red line: all contigs, green line: *E. ancora* contigs, blue line: extracted Symbiodiniaceae contigs. **c** Proportions of contigs from *E. ancora*, Symbiodiniaceae, and other symbiotic organisms (other contigs) in the initial whole holobiont transcriptome assembly. Only extracted *E. ancora* contigs (56.7%) were used for further analysis

Since the initial transcriptome assembly contained contigs from *E. ancora* gonads, symbiotic dinoflagellates (Symbiodiniaceae), and other organisms (e.g., bacteria), we first bioinformatically identified possible *E. ancora* contigs prior to detailed analyses (Fig. 2a). All assembled contigs were aligned to available genome databases of 4 scleractinian species (*Acropora digitifera*, *Pocillopora damicornis*, *Stylophora pistillata*, and *Orbicella faveolata*) and transcriptomic databases of 6 Symbiodiniaceae (*Symbiodinium* sp. A1, *Symbiodinium* sp. A2, *Breviolum* sp. B2, *Breviolum muscatinei*, Uncultured *Cladocopium* sp. and Uncultured *Durusdinium* sp.) (For more details, see Additional file 6), and contigs unambiguously matched to coral genomic databases (72,238 contigs) and to Symbiodiniaceae transcriptomic databases (31,353 contigs) were separated (Fig. 2a). Contigs matching both databases (43,332 contigs) were further aligned to the combined databases of coral genomes and Symbiodiniaceae transcriptomes, and were separated into coral contigs (23,742 contigs) and symbiotic dinoflagellate contigs (19,590 contigs) based on top hit results of BLASTN (−evalue 1e-3). Eventually, 95,980 contigs were assigned as *E. ancora*, and 50,943 to Symbiodiniaceae (Fig. 2a). *E. ancora* contigs had a GC peak at 41.5%, while symbiotic Symbiodiniaceae peaked at 50.6% (Fig. 2b). These GC contents correspond well to previous genomic studies of corals and *Breviolum minutum* [31–33].

**Fig. 3:**
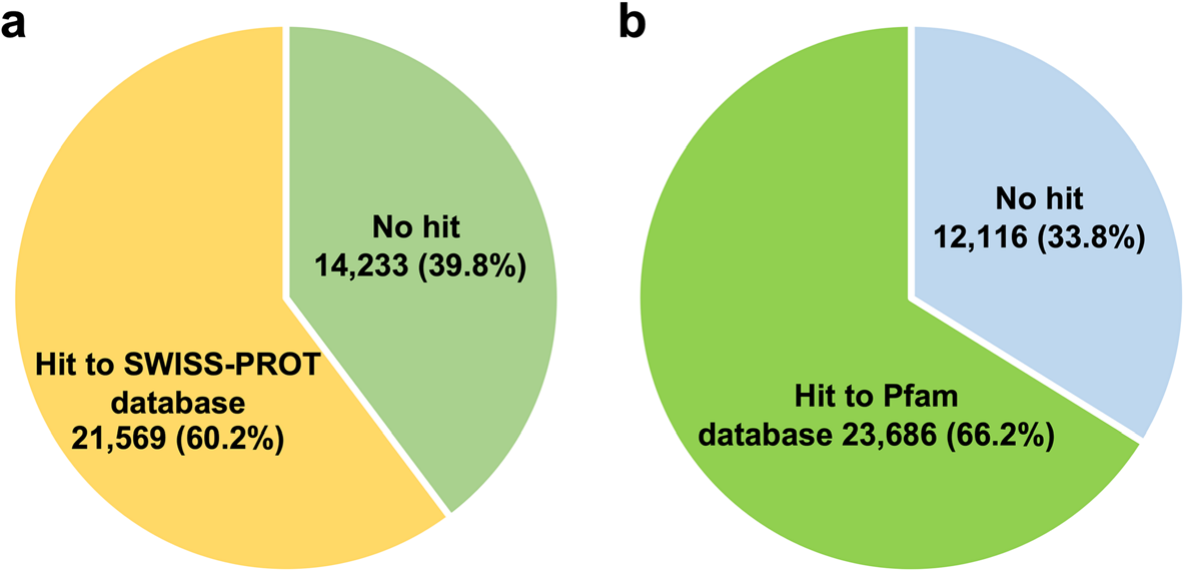
Contig numbers in the reference *E. ancora* gonadal transcriptome that matched SWISS-PROT and Pfam databases. **a** Results of BLAST searches against the SWISS-PROT database (cut-off -evalue le-5). Note that 21,569 out of 35,802 contigs (60.2%) had significant similarities with database sequences. **b**Identification of protein domains using the Pfam database (cut-off -evalue le-5) for contigs from the reference *E. ancora* gonadal transcriptome. Note that 23,686 out of 35,802contigs (66.2%) had conserved protein domains

In order to remove sequence heterogeneity originating from different individuals or different haplotypes in the same individual, translated sequences of the extracted 95,980 *E. ancora* contigs were further clustered using CD-HIT with 95% amino acid sequence identity. Finally, 35,802 contigs totaling approximately 125 Mbp (N50, 5019 bp) were used as the reference *E. ancora* gonadal transcriptome with bench-marking universal single-copy orthologs (BUSCO) of more than 90% (Table 2), which covers all *E. ancora* candidate genes involved in gametogenesis. BLAST search (BLASTP, −evalue 1e-5) revealed that 21,569 of 35,802 (60.2%) contigs had significant similarities to sequences in the SWISS-PROT database (Fig. 3a). Moreover, 23,686 of 35,802 (66.2%) contigs matched conserved protein domains in the Pfam database (Fig. 3b).

The reference *E. ancora* gonadal transcriptome contained reproduction-related genes identified in our previous studies using degenerate PCR or cDNA libraries (Additional file 7). Furthermore, evolutionarily conserved genes associated with germline development (*Gcl, Mago, Boule, and Pum1*) were identified. Genes involved in meiotic processes, such as invasion and pairing of the homologous strand *(Msh4, Msh5, Mlh1*), formation of a synaptonemal complex (*Sycp1, Sycp3*), and maintenance of chromosome structure integrity (Rad21) were also identified (Additional file 8).

### Differential gene expression analysis among different developmental stages of ovaries and testes

**Fig. 4:**
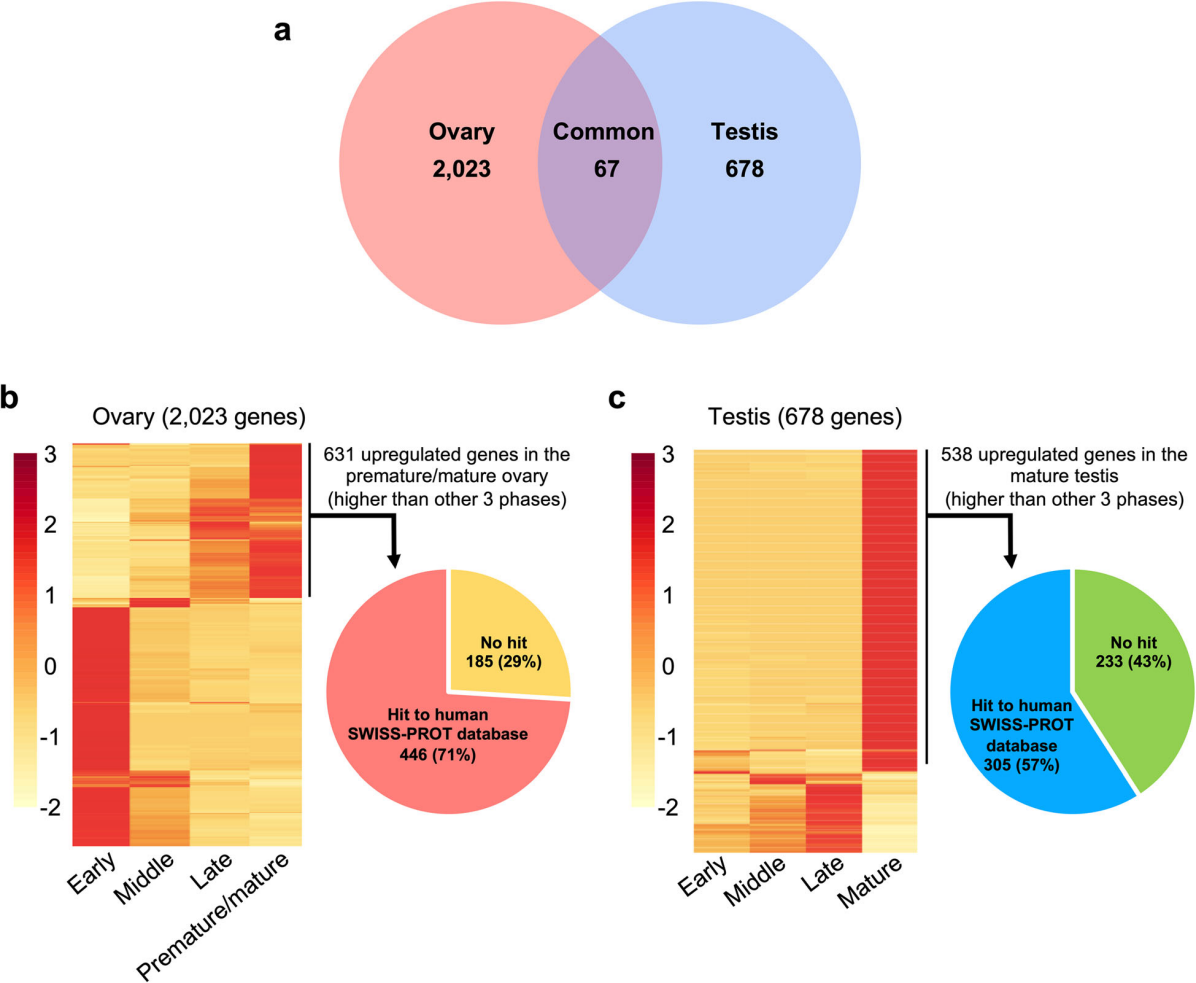
Differentially expressed genes in oogenesis and spermatogenesis of *E. ancora*. **a** 2023 and 678 genes were differentially expressed during oogenesis and spermatogenesis, respectively, and 67 of those genes were differentially expressed in both oogenesis and spermatogenesis (q-value< 0.05, ANOVA). **b** Relative gene expression levels of differentially expressed genes (2023 genes) at different phases of ovaries. CPM values were scaled to row Z-scores for each of the genes. In premature/mature ovaries, 631 genes were expressed at higher levels than in the other 3 phases. Among the 631 genes, 446 genes (71%) matched the SWISS-PROT human database, as shown in the pie chart. **c** Relative gene expression levels of differentially expressed genes (678 genes) at different phase of testes. CPM values were scaled to row Z-scores for each of the genes. In mature testes, 538 genes were expressed more highly than in the other 3 phases. Among the 538 genes, 305 (57%) matched the SWISS-PROT human database, as shown in the pie chart. In the heatmaps, each row represents a differentially expressed gene and the columns represent time points. The color bar on the left indicates expression levels

Hierarchical cluster analysis of 24 selected samples (12 testes and 12 ovaries) determined that 2 samples (Oct-female-1 and Feb-male-1) differed from all others (Additional file 9). These were assigned as outliers, possibly resulting from accidental collection of allospecific samples adjacent to the labeled colonies. To minimize data variation, the foregoing 2 samples were removed, and the remaining 22 samples were used for downstream analysis. Differential gene expression analysis of 4 developmental phases of ovaries and testes identified 2023 and 678 differentially expressed genes during oogenesis and spermatogenesis, respectively, and 67 differentially expressed genes in both ovaries and testes during gametogenesis (q-value< 0.05, Fig. 4a). There were 1165, 89, 138, and 631 upregulated genes specific to the early, middle, late, and premature/mature ovarian phases, respectively (Fig. 4b). In the testis, there were 6, 19, 115, and 538 upregulated genes specific to the early, middle, late, and mature phases, respectively (Fig. 4c).

### Upregulated genes of premature/mature ovaries

The 631 genes specifically upregulated in premature/mature ovaries were further analyzed. Four hundred forty six of those genes (71%) matched the human SWISS-PROT database (Fig. 4b). Analysis of enriched functional terms revealed that 18 GO terms were enriched in premature/mature ovaries (*P* < 0.05 and enrichment > 4-fold; Additional file 10): 16 biological processes (BP) and 2 molecular functions (MF). Of the enriched BP terms, terms related to neuronal activity such as *positive regulation of synaptic transmission, GABAergic* (GO: 0050806), *calcium ion-regulated exocytosis of neurotransmitter* (GO: 0048791), *neurotransmitter transport* (GO:0006836) and *neuronal action potential* (GO: 0019228) were highly enriched. Among enriched MF terms, *extracellular ligand-gated ion channel activity* (GO: 0005230) was most enriched (Additional file 10).

In premature/mature ovaries, genes upregulated > 5-fold more than in the other three phases (log2, FDR < 0.05), 9 genes, including those encoding GFP-like fluorescent chromoprotein, neurogenic locus notch homolog protein 3, carbonic anhydrase 2, octopamine receptor beta-1R, beta-1,4-galactosyltransferase galt-1 were identified. One of the 9 genes could not be annotated (Table 3).

**Table 3:** Genes highly upregulated in premature/mature ovaries and mature testes compared to the other 3 phases

Gonad	Annotation	Assembly ID	E-value
Ovaries (log_2_>5)	GFP-like fluorescent chromoprotein	CL9557.Contig3_All	4.00E-101
	Neurogenic locus notch homolog protein 3	CL2173.Contig10_All	1.00E-63
	Carbonic anhydrase 2	CL8194.Contig1_All	3.00E-50
	Octopamine receptor beta-1R	CL4990.Contig1_All	1.00E-22
	Beta-1,4-galactosyltransferase galt-1	Unigene16679_All	1.00E-13
	Polcalcin Juno 2	CL7546.Contig1_All	2.00E-12
	Transmembrane protein 26	Unigene179326_All	7.00E-10
	Integrin-linked protein kinase	Unigene80860_All	1.00E-08
Testes (log_2_>8)	Creatine kinase S-type, mitochondrial	CL3023.Contig3_All	0
	Creatine kinase, flagellar	CL12240.Contig1_All	0
	Omega-6 fatty acid desaturase, endoplasmic reticulum isozyme 1	Unigene27219_All	4.00E-114
	Glutamate receptor ionotropic, kainate 2	Unigene8664_All	1.00E-105
	Testis-specific serine/threonine-protein kinase 4	CL327.Contig1_All	2.00E-91
	Fibrillin-2	CL1782.Contig1_All	8.00E-71
	Monocarboxylate transporter 10	CL1025.Contig1_All	4.00E-66
	Latent-transforming growth factor beta-binding protein 1	CL10775.Contig3_All	4.00E-54
	Uncharacterized protein KIAA0895-like	CL11131.Contig2_All	1.00E-44
	Disheveled-associated activator of morphogenesis 1	CL7734.Contig1_All	2.00E-39
	BTB/POZ domain-containing protein 8	Unigene35893_All	1.00E-33
	Cyclic nucleotide-binding domain-containing protein 2	CL4865.Contig1_All	8.00E-23
	F-box/LRR-repeat protein 14	Unigene33934_All	7.00E-13
	Testis-expressed protein 11	CL11981.Contig40_All	1.00E-12
	Nuclear receptor corepressor 2	CL7171.Contig3_All	8.00E-10
	Netrin receptor UNC5C	CL47.Contig4_All	2.00E-09
	cAMP-dependent protein kinase regulatory subunit	Unigene2733_All	7.00E-09
	Polysialoglycoprotein	Unigene16171_All	3.00E-07

**Fig. 5:**
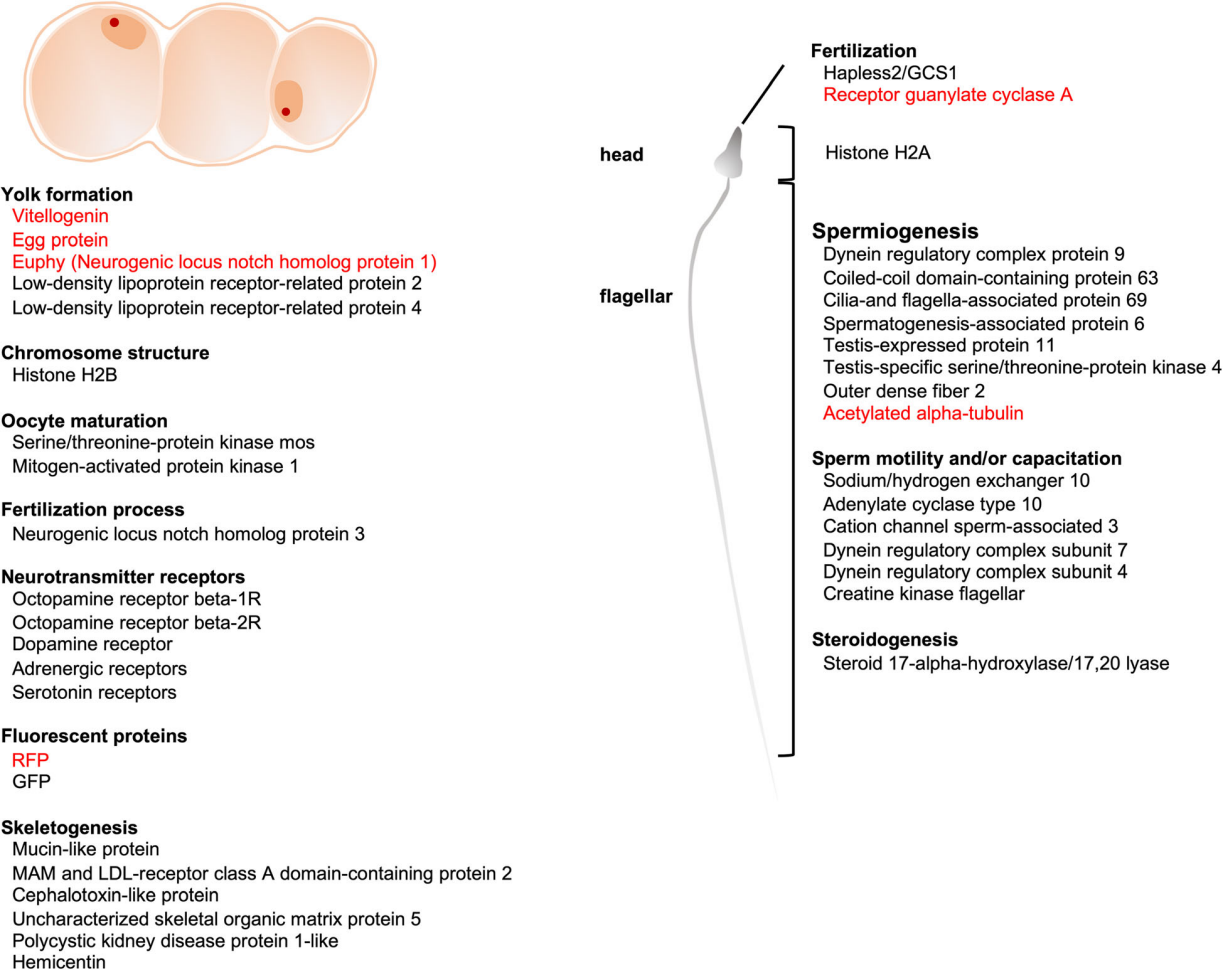
Genes potentially involved in oocyte development and maturation, and in sperm motility/capacitation, and fertilization in *E. ancora*. Genes indicated in red have been reported in our previous studies

Evolutionarily conserved genes associated with oocyte development (vitellogenin-A2, low-density lipoprotein receptor-related proteins), formation of chromosome structure (histone H2B), and oocyte maturation (serine/threonine-protein kinase mos, mitogen-activated protein kinase 1) were identified (Table 4). Additionally, several sequences similar to components of skeletal organic matrix proteins of scleractinians (mucin-like protein, MAM and LDL-receptor class A domain-containing protein 2, cephalotoxin-like protein, uncharacterized skeletal organic matrix protein 5, polycystic kidney disease protein 1-like, and hemicentin) were also identified (Table 4, Fig. 5).

**Table 4:** Upregulated genes of interest in premature/mature ovaries showing similarities to oocyte development/maturation-related genes in other animals

Category	Annotation	Assembly ID	Reference
Oocyte development	Vitellogenin-A2	CL4556.Contig6_All	(*E. ancora* Vitellogenin) [12]
	Uncharacterized skeletal organic matrix protein 5	Unigene22577_All	(*E. ancora* Egg protein) [12]
	Neurogenic locus notch homolog protein 1	Unigene28293_All	(*E. ancora* Euphy)[13]
	Low-density lipoprotein receptor-related protein 2	Unigene39647_All	[39, 40]
	Low-density lipoprotein receptor-related protein 4	Unigene20580 All	[39, 40]
Chromosome structure	Histone H2B	Unigene6592_All	[119]
Oocyte maturation	Serine/threonine-protein kinase mos	Unigene58091_All	[44]
	Mitogen-activated protein kinase 1	CL13032.Contig3_All	[44]
Neurotransmitter receptors	Octopamine receptor beta-1R	CL4990.Contig1_All	[57, 58]
	Octopamine receptor beta-2R	Unigene7363_All	[57, 58]
	Dopamine receptor	Unigene35882_All	[59]
Skeletogenesis	Mucin-like protein	CL7569.Contig3_All	[41]
	MAM and LDL-receptor class A domain-containing protein 2	CL3169.Contig2_All	[41]
	Cephalotoxin-like protein	Unigene22179_All	[41]
	Uncharacterized skeletal organic matrix protein 5	Unigene22577_All	[41]
	Polycystic kidney disease protein 1-like	CL5263.Contig3_All	[42]
	Hemicentin	CL1601.Contig3_All	[43]

### Upregulated genes of mature testes

There were 538 specifically upregulated genes in mature testes. Of those, 305 (57%) matched human genes in the SWISS-PROT database (Fig. 4c). GO functional enrichment analysis showed that 32 GO terms were enriched (*P* < 0.05 and > 4-fold enrichment; Additional file 11): 21 biological processes (BP), 8 cellular components (CC), and 3 molecular functions (MF). Of the enriched BP terms, *response to corticosteroid* (GO: 0031960), *sequestering of TGF beta in extracellular matrix* (GO:0035583), and r*egulation of cellular response to growth factor stimulus* (GO: 0090287) were highly enriched. The term *spermatid development* (GO: 0007286) was also identified, and further queries of genes representative of the term identified genes encoding testis-specific serine/threonine-protein kinases, outer dense fiber protein 2, alstrom syndrome protein 1, radial spoke head 1 homolog, and dynein regulatory complex protein 9. Of the enriched CC terms, *microfibril* (GO: 0001527) was among the most enriched. Of the 3 MF terms identified, the term *extracellular matrix structural constituent* (GO: 0030021) was most enriched (Additional file 11).

Among significantly upregulated genes in mature testes (log_2_ > 8-fold change compared to the other 3 phases, FDR < 0.05), we identified 28 genes, including those encoding creatine kinase, S-type mitochondrial, creatine kinase flagellar, omega-6 fatty acid desaturase, glutamate receptor ionotropic, kainate 2, testis-specific serine/threonine-protein kinase 4, and fibrillin-2 (Table 3). Ten of the 28 genes could not be annotated.

Evolutionarily conserved genes involved in spermiogenesis and fertilization were further explored among upregulated genes in mature testes (Table 5). We identified a number of important genes encoding proteins associated with spermiogenesis (spermatogenesis-associated protein 6, cilia- and flagella-associated protein 69), sperm motility and/or capacitation (dynein regulatory complex subunit 7, sodium/hydrogen exchanger 10, creatine kinase, flagellar, adenylate cyclase type 10, and cation channel sperm-associated protein 3), and fertilization process (hapless 2/generative cell specific 1 and receptor guanylate cyclase). We also identified a gene encoding steroid 17α-hydroxylase/17,20-lyase (Cyp17a), a key enzyme in sex steroid and cortisol production (Fig. 5, Table 5).

**Table 5:** Upregulated genes of interest in mature testes showing similarities to sperm-related genes in other animals

Category	Annotation	Assembly ID	Reference
Spermiogenesis	Spermatogenesis-associated protein 6	Unigene40770_All	[76]
	Cilia- and flagella-associated protein 69	Unigene30111_All	[77]
	Testis-specific serine/threonine-protein kinase 1	CL2440.Contig1_All	[78, 79]
	Testis-specific serine/threonine-protein kinase 2	Unigene8363_All	[78, 79]
	Outer dense fiber protein 2	CL11766.Contig4_All	[80]
	Alstrom syndrome protein 1	CL2440.Contig1_All	[81]
	Radial spoke head 1 homolog	CL2440.Contig1_All	[82]
	Dynein regulatory complex protein 9	CL4871.Contig6_All	[83]
Sperm motility/capacitation	Dynein regulatory complex subunit 7	CL8060.Contig33_All	[86]
	Sodium/hydrogen exchanger 10	CL11505.Contig1_All	[87]
	Creatine kinase, flagellar	CL12240.Contig1_All	[88]
	Adenylate cyclase type 10	CL1089.Contig3_All	[87]
	Cation channel sperm-associated protein 3	Unigene27629_All	[87]
	Cyclin-F	Unigene55093_All	[89]
	Dynein regulatory complex subunit 4	Unigene7541_All	[90]
Chromosome structure	Histone H2A	CL4659.Contig4_All	[120]
Fertilization process	Hapless 2	CL1879.Contig3_All	[109, 110]
	Receptor guanylate cyclases	CL4659.Contig4_All	[22]
	Cyclic nucleotide-gated channel cone photoreceptor subunit alpha	CL3457.Contig2_All	[153]
Steroidogenesis	Steroid 17-alpha-hydroxylase/17,20 lyase	Unigene38823_All	[104]

## Discussion

### Scleractinian gonadal transcriptome assembly

Since scleractinian gametogenesis occurs exclusively in gonads, isolated gonads (but not whole polyps) are useful to explore genes associated with gametogenesis. However, gonad isolation is technically difficult in many scleractinians due to small polyp sizes. Gonad isolation not only requires an understanding of polyp anatomy, but also technical skill. The present study applied previously established techniques for gonad isolation from *E. ancora* polyps [12] to the current transcriptomic study. Bioinformatics methods to eliminate contigs from symbiotic dinoflagellates or other contaminants were also employed [33]. 60.2% of the contigs in the *E. ancora* gonadal transcriptome assembly showed similarities to entries in the SWISS-PROT database (Fig. 3). Specifically, 68% were similar to *Stylophora pistilata* gene models [34] and 59.7% to *Pocillpora damicornis* gene models [35] in SWISS-PROT. In addition, conserved Pfam protein domains were detected in 66.2% of contigs in the *E. ancora* gonadal transcriptome (Fig. 3). Conserved Pfam protein domains were detected in 54% of sequences in the *Heliopora coerulea* transcriptome assembly [36]. Other transcriptome assemblies showed similar percentages: *Dendrophyllia* sp. (48.8%), *Eguchipsammia fistula* (45.4%), and *Rhizotrochus typus* (51.3%) [37]. The *E. ancora* gonadal transcriptome is clearly comparable to other coral genomic or transcriptomic datasets. The present transcriptome assembly allowed us to identify sex-specific and gonadal phase-specific upregulated genes as well as evolutionarily conserved genes associated with germ cell development. The resulting dataset will provide a foundation for future research investigating molecular and cellular mechanisms of gametogenesis in scleractinians.

### Characteristics of premature/mature ovaries as assessed by anatomical and histological analyses

The observed growth of oocytes and the loss of germinal vesicles in oocytes of premature/mature ovaries suggest that oocytes were still actively accumulating essential materials (e.g., yolk and other components) for survival and development of embryos until just before maturation. Also, the oocyte maturation process, including germinal vesicle breakdown (GVBD) and resumption of meiosis occurred in some oocytes.

### Upregulated genes in premature/mature ovaries

Yolk formation and accumulation is one of the most important aspects of oogenesis for oviparous animals. In scleractinian eggs, several major yolk proteins, including vitellogenin (Vg), a female-specific phosphoglycolipoprotein, and large amounts of lipids (e.g., wax esters, fatty acids, phosphatidylethanolamines, and phosphatidylcholines) have been identified to date [12, 13, 19, 20, 38]. The present study found that transcripts encoding 3 major yolk proteins were upregulated (Vg, Egg protein, and Euphy, Fig. 5, Table 4), in agreement with histological observations, indicating that oocytes were actively accumulating yolk materials. Those yolk proteins are produced by ovarian somatic cells adjacent to oocytes [12, 13]. However, little is known about the uptake mechanisms of yolk proteins by oocytes. Although receptor-mediated endocytosis has been hypothesized, related receptor molecules have not been identified yet [12]. The present study also identified transcripts encoding two types of low-density lipoprotein receptor-related proteins (Lrps) as upregulated genes in premature/mature ovaries (Fig. 5, Table 4). In some teleosts, a member of Lrps, Lrp13, serves as one of the Vg receptors expressed on oocyte membranes [39, 40]. Thus, the Lrps identified here may be involved in uptake mechanisms of yolk materials in scleractinians, and are promising candidate receptors for Vg and/or other lipoproteins in future studies.

In addition to the major yolk materials, eggs of scleractinians are assumed to accumulate materials essential for larval development. Among the upregulated genes in premature/mature ovaries, we identified several sequences similar to components of skeletal organic matrix proteins found in *A. digitifera* [41], *A. millepora* [42], and *S. pistillata* [43]. Since no skeleton formation occurs in ovaries, it is likely that these gene products (mRNA and proteins) are stored in oocytes during oogenesis to be used for skeleton formation during larval development. We cannot rule out the possibility that the identified genes may have other functions in oocyte development/maturation.

The occurrence of GVBD in some oocytes of ovaries collected in April 2017 was an unexpected finding, because mature gametes were not observed in testes collected at the same time. It is possible that timing of oocyte maturation was split among oocytes and/or ovaries over April and May (or June) for unknown reasons. We cannot completely rule out the possibility that the GVBD was partially induced by isolation of ovaries from polyps, i.e., mechanical stress. Nevertheless, this study successfully identified two sequences similar to the serine/threonine-protein kinase mos (Mos) gene and the mitogen-activated protein kinase 1 (Mapk1) gene, which contribute to signaling pathways of oocyte maturation in a variety of animals, including cnidarians [44] (Fig. 5). Upregulation of these two genes in premature/mature ovaries implies that they may also function in oocyte maturation in scleractinians. Previous studies regarding oocyte maturation in scleractinians were limited to histological observations and focused on the presence and timing of GVBD [45, 46]. To the best of our knowledge, this is the first study to identify these candidate molecules in oocyte maturation of scleractinians.

In a variety of animals, hormones (i.e., steroids, growth factors, peptides, and other substances) are involved in the reproduction [47–54]. In *Acropora* species, transcriptomic studies suggest that melanopsin-like homolog and /or neuropeptides [55] and Rhodopsin-like receptors [56] are involved in the signaling pathway for spawning in scleractinians. Enriched BP terms in *E. ancora* premature/mature ovaries imply that neuronal activity is significantly higher than during other phases. Upregulation of transcripts similar to genes encoding monoamine receptors (e.g., octopamine receptors, dopamine receptors, adrenergic receptors, and serotonin receptors, [57, 58] Fig. 5, Table 4) also support this assumption. Recent studies show that some neurotransmitters (dopamine and serotonin) are also involved in regulation of scleractinian spawning. Treatment of *Acropora tenuis* with dopamine during the final phase of gametogenesis inhibited spawning [59]. By contrast, treatments with serotonin and its precursor, L-5-hydroxytryptophan (5-HTP) induced spawning of *Acropora cervicornis* [60]. Taking all these lines of evidence into account, the identified monoamine receptors may also be essential during the premature/mature phase of *E. ancora* oogenesis. It will be of interest to investigate whether treatment of female *E. ancora* with these neurotransmitters induces or inhibits oocyte maturation and spawning.

Of particular interest is the upregulation of three genes encoding neurogenic locus notch homolog proteins in premature/mature ovaries (Additional file 12). The Notch signaling pathway is conserved across animal taxa, and regulates cell-cell interactions and cell fate determination [61]. One of the identified genes, neurogenic locus notch homolog protein 1, encodes Euphy, a novel major yolk protein in *E. ancora* oocytes identified in our previous study [13]. The remaining 2 genes have not been previously reported. Although both sequences possess EGF-like domain repeats typifying notch homolog proteins, they are structurally distinct from Notch1 identified in vertebrates (e.g., human Notch1). These may be novel genes that emerged after gene duplication, domain shuffling, and rapid molecular evolution in cnidarian/scleractinian lineages [41, 42]. Interestingly, one of them, neurogenic locus notch homolog protein 3, was highly and significantly upregulated, and contains a zona pellucida (ZP) protein and transmembrane domains (Fig. 5, Additional file 12). The ZP is the extracellular matrix (ECM) surrounding mammalian oocytes, composed of four glycoproteins (ZP1-ZP4). ZP functions during oogenesis, fertilization, and preimplantation development in mammals [62]. In jellyfish, a ZP domain-containing protein called mesoglein, which resembles mammalian ZP, was identified in the contact plate of oocytes [63]. Although scleractinian oocytes have neither a protective coat nor a membrane surrounding them, this finding implies that the identified ZP domain-containing protein probably participates in oogenesis and subsequent fertilization processes.

GFP is one of the natural pigments of corals [64–67]. Although the natural functions of GFP remain obscure, proposed functions include photoprotection from high UVA/blue irradiation, photosynthetic enhancement, phototaxis of zooxanthellae [68–72], and antioxidant activity [73, 74]. We previously showed that *E. ancora* oocytes express an endogenous RFP with H_2_O_2_ degradation activity from early to mature stages of oocytes, and suggested a possible role of RFP in protecting oocytes from oxidative stress during oogenesis [15]. Our finding implies that not only RFP, but also GFP may serve in oogenesis, particularly during the premature/mature phase (Fig. 5).

### Characteristics of mature testes as assessed by histological and cytological analyses

Spermiogenesis is a process by which haploid spermatids undergo a complex series of morphological changes, and eventually become elongated functional sperm. The presence of spermaries having both round spermatids and mature sperm in testes collected in June 2017 suggested that spermiogenesis was occurring in the testes at the time of collection, and that genes involved in regulation of spermiogenesis were being expressed in testes.

### Upregulated genes in mature testes

Morphological changes of male germ cells during spermiogenesis include flagellum formation, nuclear DNA condensation, and elimination of organelles and cytoplasm. Scleractinian spermiogenesis is generally morphologically similar to that of vertebrates, except that male germ cells possess long flagella from early to late stages of development [21]. Nevertheless, scleractinian male germ cells possess typical flagellar axonemes, characterized by a“9 + 2” arrangement of microtubules [21, 75]. In this study, further queries of genes associated with *spermatid development* (GO term:0007286), together with literature-based gene identification, allowed us to identify various candidate genes encoding proteins of flagellar components [76–83]. The presence of a conserved molecular toolkit for spermiogenesis suggests that scleractinians and vertebrates share similar characteristics at both morphological and molecular levels.

Sperm motility is important for most scleractinians, which fertilize externally in seawater. Sperm of acroporid corals remain completely immotile in seawater until they come close to eggs, whereupon they acquire motility [84]. The presence of chemoattractants and involvement of intracellular pH elevation and Ca^2+^-dependent signal transduction in sperm motility have been experimentally demonstrated [84, 85]. Molecules regulating flagellar motility still remain largely unexplored in scleractinians. This study identified a number of important genes encoding proteins involved in sperm motility and/or capacitation in mammals and sea urchins, such as cation channel sperm-associated protein 3 (CatSper3), sodium/hydrogen exchanger (sNHE), and adenylate cyclase type 10 (sAC) [86–90] (Fig. 5, Table 5). These findings support the hypothesis of Romero and Nishigaki that CatSper3, sNHE, and sAC form prototypical machinery for sperm flagellar beating in metazoans [87]. This study further identified the gene encoding creatine kinase, flagellar, which was first identified from flagella of sea urchin sperm, participating in energy transport from sperm heads to the flagella during sperm motility [89]. Genes associated with sperm motility and/or capacitation in scleractinians suggest that these features were most likely present in the common ancestor prior to divergence of the cnidarian and bilaterian lineages.

Sex steroids are critical for sex differentiation, gametogenesis, and gamete maturation in vertebrates [91–95]. Sex steroids (e.g., estrogen, testosterone, and progesterone) have been demonstrated in several scleractinians, including *E. ancora* [96–99]. Additionally, the correlation between sex steroid levels and gametogenic cycles has led to the hypothesis that sex steroids may be involved in regulation of scleractinian reproduction [97, 99]. Steroid biosynthesis is catalyzed by various steroidogenic enzymes. Although steroid biosynthetic activities are known from extracts of some scleractinian tissues [97, 98, 100–102], only one gene encoding a steroidogenic enzyme, 17β-hydroxysteroid dehydrogenase type 14 (17β-hsd 14), has been identified and characterized so far [103]. In the present study, a gene encoding steroid 17α-hydroxylase/17,20-lyase (Cyp17a) (Fig. 5, Table 5), a key enzyme in production of sex steroids and cortisol [104], was upregulated in mature testes. Although further analysis is required to clarify its activity, the presence of this enzyme implies that steroid biosynthesis occurs in mature testes, and the produced sex steroids/cortisol could be associated with maturation of male germ cells in scleractinians.

Molecules involved in fertilization remain largely unknown in scleractinians. We found that a gene similar to Hapless 2/Generative Cell Specific 1 (Hap2/Gcs1) was upregulated in mature testes (Fig. 5, Table 5). Hap2/Gcs1 was first identified as a male gamete-specific transmembrane protein in lilies [105]. The coding gene is found in genomes of most major eukaryotic taxa (e.g., protozoa, plants, and animals) except fungi [106, 107]. Functional analysis with the mutant/gene targeting system showed that Hap2/Gcs1 are essential for gamete fusion in *Arabidopsis* [105], the protozoan parasite, *Plasmodium* [106], and the green alga, *Chlamydomonas* [108]. Expression of Hap2/Gcs1 was also confirmed in male germ cells of some cnidarians, such as *Hydra* [109] and the starlet sea anemone, *Nematostella vectensis* [110], and its involvement in fertilization has been demonstrated in sea anemones [110]. Upregulation of Hap2/Gcs1 in *E. ancora* mature testes suggests that Hap2/Gcs1 participates in scleractinian sperm-egg fusion. Most recently, we reported that receptor guanylate cyclase A (rGC-a) (also known as atrial natriuretic peptide receptor 1 in mammals) is expressed in *E. ancora* sperm flagella [22] (Fig. 5, Table 5). rGCs are expressed on sperm and serve as receptors for egg-derived sperm-activating and sperm-attracting factors in some echinoderms and mammals [111–114]. Taken together, evolutionarily conserved proteins underlie fertilization mechanisms of scleractinians.

### Other major findings and potential applications

Genes encoding Histone H2B and Histone H2A were upregulated in premature/mature ovaries and mature testes, respectively (Fig. 5, Table 4, 5). Histones are the major protein components of chromatins in eukaryote cell nuclei. Five histone protein families exist: the core histone families (H2A, H2B, H3, and H4) and the linker histone family (H1) [115]. Core histones are components of the nucleosome core, whereas linker histones are present in adjacent nucleosomes, where they bind to nucleosomal core particles, and stabilize both nucleosome structure and higher-order chromatin architecture [115, 116]. Various isoforms of each family have been identified as histone variants, and their importance in diverse cellular processes (e.g., transcriptional control, chromosome segregation, DNA repair and recombination, and germline specific translational regulation) have been revealed [117–120]. In scleractinians, although sequences of the histone gene cluster have been identified in *Acropora formosa* (H3, H4, H2A, and H2B) [121] and *Acropora gemmifera* (H3, and H2B) [122], differences in gene expression levels between ovaries and testes have not been reported so far. This study revealed the existence of histone variants showing sexually dimorphic expression in scleractinians. In the cnidarian model organism, *Hydractinia echinata*, 19 genes encoding histones were identified, and some of them, such as histone H2A.X and five H2B variants, are specifically expressed in female and male germ cells, respectively [123]. Our findings imply that the identified histone may control gene expression in female and male germ cells during scleractinian gametogenesis.

Studies of a variety of animals have revealed that a set of specialized and highly conserved genes govern germline specification, development, meiosis, and/or maintenance in metazoans [124–136]. In the gonadal transcriptome, we could identify many genes associated with germline specification and meiotic processes (Additional file 8). Although further spatiotemporal expression analyses and functional assays are required to clarify their functions, their expression in gonads implies that these genes participate in scleractinian germline development and meiosis.

The *E. ancora* gonadal transcriptome assembly includes a large number of genes without homology to sequences in the SWISS-PROT database. These findings suggest that although scleractinian gametogenesis shares many common molecular characteristics with gametogenesis in other metazoans, it also possesses characteristics that developed in evolutionarily unique ways. Further characterization and functional studies of these unannotated genes will clarify unique features in scleractinian gametogenesis, and this will eventually lead to comprehensive understanding of scleractinian gametogenesis.

The knowledge obtained in the present study will be useful for ecological studies and coral aquaculture. For instance, since scleractinian corals have no secondary sexual characteristics, histological analysis has traditionally been used to investigate polyp or colony sex, as well as to determine the status of germ cell development. However, histological analysis of scleractinians is time consuming. It generally requires decalcification steps, and the whole histological process sometimes takes 1–2 weeks. Identification of molecular markers for determining colony sex and germ cell development status offers a useful alternative process. Colony sex and germ-cell type could be determined faster using PCR with markers, than by histological means. Sex- and gonad phase-specific genes identified in this study would be candidates.

## Conclusions

Analysis of upregulated genes in premature/mature gonads allowed us to identify many genes potentially involved in oocyte development, oocyte maturation, spermiogenesis, sperm motility/capacitation, and fertilization processes (Fig. 5). We identified a large number of sex-biased or sex-specific genes and shed light on possible molecular mechanisms of scleractinian gametogenesis, which appear to be coordinated by both conserved and novel genes. This study and its generated datasets thus provide a foundation for future studies regarding gametogenesis and differences between sexes from molecular and cellular perspectives. Furthermore, our transcriptome assembly will be a useful reference for future development of sex-specific and/or stage-specific markers for germ cells for use in coral aquaculture and ecological studies.

## Methods

### Sample collection

*E. ancora* specimens were collected by scuba divers at Nanwan Bay, Kenting National Park, in southern Taiwan (21°57′N, 120°46′E). Approximately 10 colonies were labeled, and gonads (> 20 gonads) of labeled colonies were microscopically isolated at different times during a 9-month period from October 2016 (non-spawning period) to June 2017 (spawning period) (Fig. 1d). Collection was approved by the administrative office of Kenting National Park (issue number: 1010006545). For RNA-seq, collected samples were snap frozen in liquid nitrogen, and stored at − 80 °C until use. Some of the isolated gonads were also fixed with 20% Zinc-Formal-Fixx (Thermo Fisher Scientific, Pittsburgh, PA, USA) for histological analysis. Experiments were performed in accordance with principles and procedures approved by the Institutional Animal Care and Use Committee, National Taiwan Ocean University, Taiwan.

### Histological analysis for sample selection

Histological analyses were performed to determine developmental phases of gonads, and to select samples for RNA-seq. Isolated gonads (> 10 gonads/colony/time point) were analyzed according to the methodology in our previous studies [8, 9]. Developmental stages of germ cells were determined according to previous criteria [8, 9] with some modifications (see Table 1).

### RNA extraction and RNA-seq library construction

In total, 12 testes and 12 ovaries (3 colonies × 4 time points) were selected based on the results of histological analyses. Total RNA of the 24 samples was extracted using TRIzol reagent (Thermo Fisher Scientific) according to the manufacturer’s protocol. DNase I treated-RNA samples were sent to Beijing Genomics Institute (BGI, Shenzhen). RNAs were qualified using a Bioanalyzer 2100 (Agilent Technologies, Palo Alto, CA, USA) with an RNA 6000 labchip kit (Agilent Technologies) and all samples were confirmed as high-quality RNA (RIN > 8). Twenty four RNA-seq libraries were constructed using TruSeq mRNA Library Prep Kits v2 (Illumina, San Diego, CA, USA), and sequenced with 150-bp paired-ends (150PE) on an Illumina HiSeq X Ten. Illumina adaptors, low-quality sequences (Phred value Q < 20), and reads with a high proportion of N (> 5%) were removed. Cleaned sequencing data were used for subsequent analyses.

### De novo assembly and annotation of the *E. ancora* transcriptome

The transcriptome assembly of the *E. ancora* “holobiont”, the host and its symbiotic organisms, was created by BGI as follows. Clean reads of 24 individual samples were assembled de novo using Trinity v2.0.6 software [137] (parameter settings: –min_contig_length 150 –CPU 8 –min_kmer_cov 3 –min_glue 3 –bfly_opts ‘-V 5 –edge-thr = 0.1) and assembled sequences were clustered using Tgicl v2.0.6 software [138] (parameter settings: -l 40 -c 10 -v 25 -O ‘-repeat_stringency 0.95 -minmatch 35 -minscore 35′). Since gonadal samples contained substantial numbers of symbiotic dinoflagellate cells, we bioinformatically separated sequences originating from *E. ancora*, algal symbionts (Symbiodiniaceae), or microbes as follows. All assembled sequences were aligned to available genomic databases of 4 scleractinian corals and 6 Symbiodiniaceae transcriptomic databases using BLASTN (−evalue 1e-3). These databases included *A. digitifera* [31, 139], *P. damicornis* [35, 140], *S. pistillata* [34], and *O. faveolata* [141], *Symbiodinium* sp. A1 [142], *Symbiodinium* sp. A2 [143], *Breviolum sp.* B2 [143], *Breviolum muscatinei* [144], Uncultured *Cladocopium* sp. [145] and uncultured *Durusdinium* sp. [145] (For more detailed information on the databases, see Additional file 6). Contigs aligned exclusively to the coral genome database were annotated as “*E. ancora* contigs”, while those that aligned only to Symbiodiniaceae transcriptome databases were annotated as “Symbiodiniaceae contigs”. To separate contigs aligned to both the coral genome and Symbiodiniaceae transcriptomic databases, contigs were re-aligned BLASTN (−evalue 1e-3) using a combined database of coral genomes and Symbiodiniaceae transcriptomes. Based on the top hit results of BLASTN (corals or Symbiodiniaceae), contigs were annotated as “*E. ancora* contigs” or “Symbiodiniaceae contigs” (Fig. 2). All databases used in the present study were downloaded on 3/18/2019. Nucleotide sequences were again clustered using CD-HIT [146] with 97% identity for removing sequences possibly originating from different individuals or haplotypes in a single individual. Finally, contigs were translated into amino acid sequences using the longorf script [147] and clustered using CD-HIT with 95% identity. Completeness using the assembled sequences was assessed using BUSCO (bench-marking universal single-copy orthologs) version 3 [148, 149] in transcriptome mode. Reference *E. ancora* gonadal transcriptome contigs were annotated as follows: 1) BLAST searches against public protein databases: SWISS-PROT database (−evalue 1e-5) (Consortium 2011) (3/18/2019), 2) Identification of conserved protein domains with the Pfam database (−evalue 1e-5) [150, 151].

### Identification of reproduction-related genes in ovaries and testes

Genes important in metazoan reproduction were searched in the reference *E. ancora* gonadal transcriptome, based on the literature [8, 9, 12, 13, 15, 16, 21, 22, 72, 126–136, 152, 153]. Two strategies were adopted. 1) Full-length cDNA sequences of genes in vertebrates and invertebrates were retrieved from Genbank (NCBI), and local BLAST searches were conducted (BLASTP, cut-off -evalue 1e-5) against translated sequences from the reference *E. ancora* gonadal transcriptome. 2) Gene names or keyword searches for target categories were performed in SWISS-PROT annotation results.

### Differential gene expression analysis

First, possible outlier RNA-seq samples were examined by mapping raw reads to assembled sequences with Bowtie2 v2.2.6 software [154] (parameter setting: -q –phred33 –sensitive –dpad 0 –gbar 99,999,999 –mp 1,1 –np 1 –score-min L,0,-0.1 -I 1 -X 1000 –no- mixed –no-discordant -p 1 -k 200) and the mapping coverage of contigs was determined with RSEM v1.2.12 software [154] under default settings. The hclust package in R was used to perform a hierarchical cluster analysis of RNA-seq samples [155]. The above analyses were performed by BGI. Illumina adaptors and low-quality sequences (quality score > Q20, reads length > 25 bp) were removed from raw RNA sequences of the remaining samples using CUTADAPT v1.16 [156] . Using SALMON v0.13.1 [157], clean reads were mapped to the reference *E. ancora* transcriptome contigs. Further statistical analyses based on mapping counts were done using edgeR v3.24.3 [158, 159] in R software. Mapping counts were normalized by the trimmed mean of M values (TMM) method, and then converted to counts per million (CPM). Differentially expressed genes in each phase of ovaries and testes were identified using the likelihood ratio test (glmLRT). All *P*-values obtained by likelihood ratio test were adjusted with the Benjamini-Hochberg method. Genes (or transcripts) representing FDR < 0.05 were considered as differentially expressed genes. CPM values were used to identify genes that were differentially expressed in each phase of ovaries and testes, respectively (ANOVA with q-value< 0.05). For heatmap generation, CPM values were scaled to row Z-scores for each of the genes that were highly expressed in each phase of gonads.

### Gene enrichment analysis

UniProt IDs were assigned for each reference *E. ancora* gonadal transcriptome contig based on best matches against the human SWISS-PROT database with BLASTP (cutoff of -evalue 1e-5) [160]. Gene enrichment analysis of Gene Ontology (GO) was performed with the assigned UniProt ID using DAVID Bioinformatics Resources 6.8 (> 4-fold enrichment and *P* < 0.05) [161, 162]. UniPort IDs of the reference *E. ancora* gonadal transcriptome were used as a background for the DAVID analysis.

## Supplementary information

**Additional file 1 **Microscopic observation of ovaries and testes isolated in April and June 2017, respectively. **(a)** External appearances of oocytes having germinal vesicles in isolated ovaries collected in April 2017. **(b)** The external appearance of oocytes without germinal vesicles collected at the same times as the samples shown in (a). Only one oocyte has a germinal vesicle (arrow). **(c)** Cytological appearance of an isolated testis collected in June 2017. Morphologically mature sperm with triangular head shapes were observed (arrows).

**Additional file 2.** (Table) Summary of clean read data for 24 samples used in de novo assembly.

**Additional file 3.** DDBJ accession numbers for the reference gonadal transcriptome

**Additional file 4 **DDBJ accession numbers for the *E. ancora* contigs

**Additional file 5.** DDBJ accession numbers for the Symbiodiniaceae contigs of DDBJ accession number

**Additional file 6 **(Table) Reference databases used for identifying E. ancora-originated contigs from the E. ancora holobiont transcriptome assembly

**Additional file 7 **(Table) Reproduction-related genes of E. ancora identified in our previous studies

**Additional file 8 **(Table) Evolutionarily conserved genes in metazoan reproduction identified in the E. ancora gonadal transcriptome assembly

**Additional file 9 **Hierarchical Clustering analysis of *E. ancora* gonadal samples used in this study. Twelve testis samples (3 colonies, 4 time points) and 12 ovary samples (3 colonies, 4 time points) were subjected to analysis. The cluster dendrogram showed that 2 samples (Oct-female-1 and Feb-male-1) are outliers, while others belong to a similar group. The 2 outliers were removed and the 22 remaining samples were used for gene expression analysis.

**Additional file 10 **GO functional analysis of upregulated genes in premature/mature ovaries. Significantly (> 4-fold change, *P* < 0.05) enriched GO terms in biological processes (blue bar), and molecular function (yellow bar). The X-axis represents the magnitude of change. The Y-axis represents the GO functional category.

**Additional file 11.** GO functional analysis of upregulated genes in mature testes. Significantly (> 4-fold change, P < 0.05) enriched GO terms in biological processes (blue bar), cellular component (green bar), and molecular function (yellow bar). The X-axis represents the magnitude of change. The Y-axis represents the GO functional category.

**Additional file 12.** Schematic figures depicting domain structures of neurogenic locus notch homolog protein. (a) Neurogenic locus notch homolog proteins 1. (b) Neurogenic locus notch homolog proteins 2. (c) Neurogenic locus notch homolog proteins 3.

## Data Availability

Datasets created and/or analyzed in this study are available in the Sequence Read Archive (SRA) of DNA DataBank of Japan (DDBJ) under BioProject accession PRJDB9831. Transcriptome assemblies have been deposited at the DDBJ/European Nucleotide Archive/GenBank Transcriptome Shotgun Assembly (TSA) under accession numbers ICQS01000001-ICQS01169272. The datasets supporting the conclusions of this article are included within the article and its additional files (Additional files 6, 7, 8).

## Abbreviations

GVBD: Germinal vesicle breakdown
SRA: Sequence read archive
DDBJ: DNA Data Bank of Japan
bp: Base pair
GC content: Guanine-Cytosine content
Pfam: Protein family
Gcl: Germ cell-less
Mago: Mago nashi
Boule: Boule-like
Pum1: Pumilio homolog 1
Dmc1: DNA meiotic recombinase 1
Muts4: MutS protein homolog 4
Muts5: MutS protein homolog 5
Mlh1: DNA mismatch repair protein Mlh1
Sycp1: Synaptonemal complex protein 1
Sycp2: Synaptonemal complex protein 2
Sycp3: Synaptonemal complex protein 3
Rad21: Double-strand-break repair protein rad21
FDR: False discovery rate
GO: Gene ontology
BP: Biological processes
MF: Molecular functions
CC: Cellular components
SOMPs: Skeletal organic matrix proteins
Lrps: Low-density lipoprotein receptor related proteins
Lrp13: Low-density lipoprotein receptor-related-protein 13
Vg: Vitellogenin
Mos: Serine/threonine-protein kinase mos
Map k1: Mitogen-activated protein kinase 1
5-HTP: 5-Hydroxytryptophan
Notch1: Neurogenic locus notch homolog protein 1
ZP: Zona pellucida
ECM: Extracellular matrix
GFP: Green fluorescent protein
UVA: Ultraviolet A
RFP: Red fluorescent protein
CatSper3: Cation channel sperm-associated protein 3
sNHE: Sodium/hydrogen exchange
sAC: Adenylate cyclase type 10
17beta-hsd 14: 17beta-hydroxysteroid dehydrogenase type 14
Cyp17a: 17α-hydroxylase/17,20-lyase
Hap2/Gcs1: Hapless 2/Generative Cell Specific 1
rGC: Receptor guanylate cyclase
PCR: Polymerase chain reaction
BGI: Beijing Genomics Institute
RIN: RNA integrity number
PE: Paired-end
BUSCO: Bench-marking universal single-copy orthologs
CPM: Count per million
ANOVA: Analysis of variance
DAVID: Database for annotation, visualization and integrated discovery
