# Supplementary figures and images for: De novo transcriptome assembly from the gonads of a scleractinian coral, Euphyllia ancora: molecular mechanisms underlying scleractinian gametogenesis
Source: BMC Genomics. 2020 Oct 21;21:732. doi: 10.1186/s12864-020-07113-9 (PMC7579821; doi:10.1186/s12864-020-07113-9)

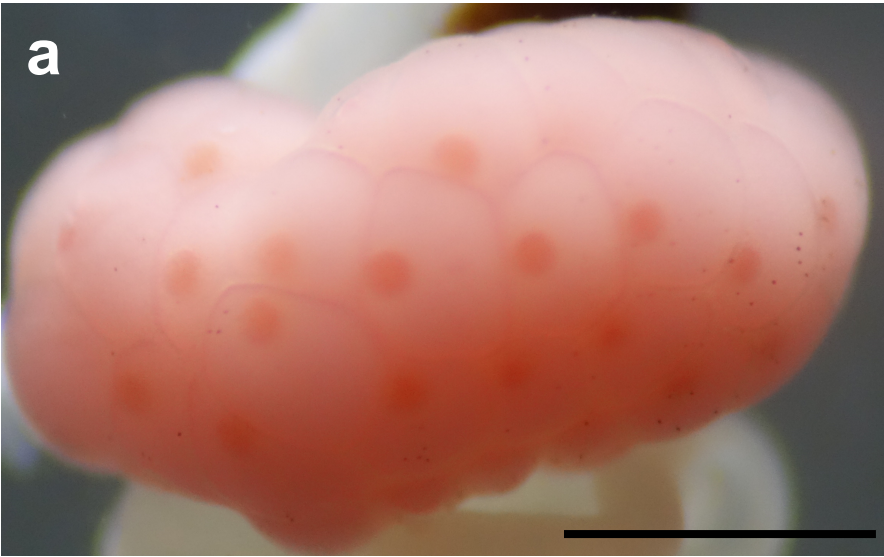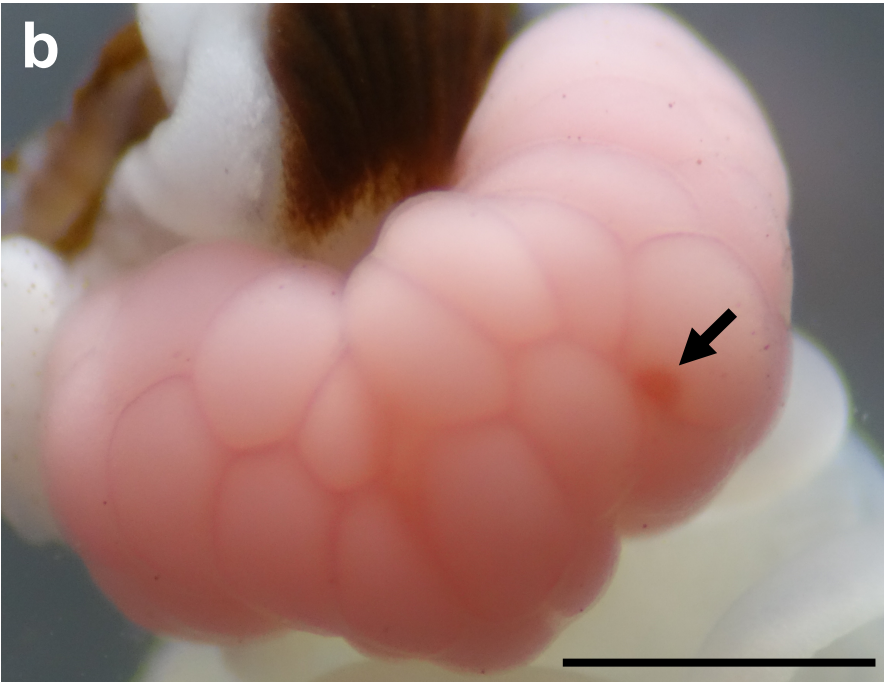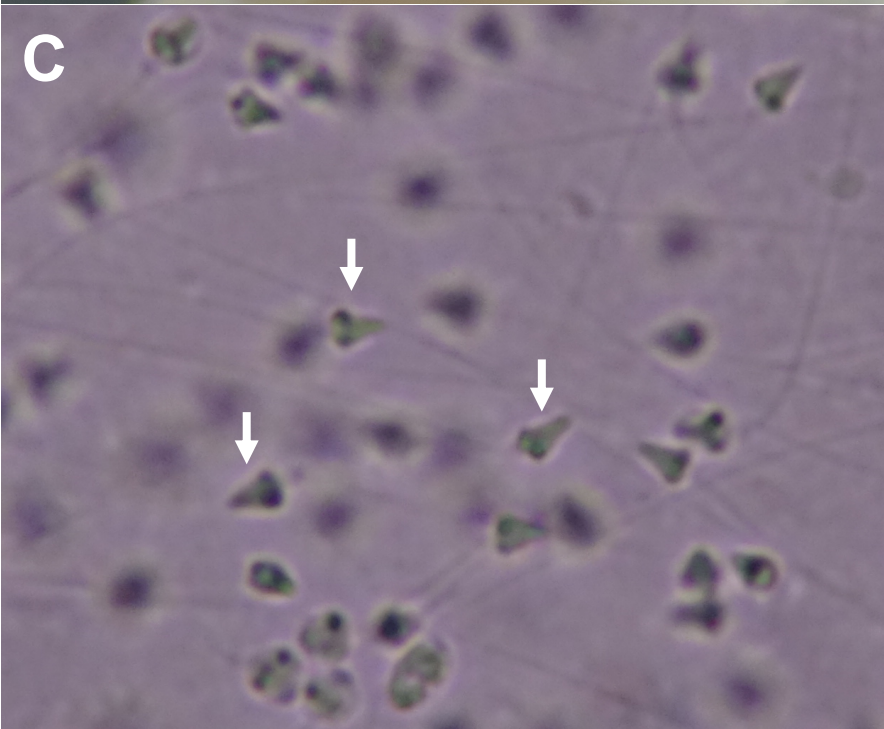

Supplement: Supplementary file 1 — Additional file 1 Microscopic observation of ovaries and testes isolated in April and June 2017, respectively. (a) External appearances of oocytes having germinal vesicles in isolated ovaries collected in April 2017. (b) The external appearance of oocytes without germinal vesicles collected at the same times as the samples shown in (a). Only one oocyte has a germinal vesicle (arrow). (c) Cytological appearance of an isolated testis collected in June 2017. Morphologically mature sperm with triangular head shapes were observed (arrows). [file 12864_2020_7113_MOESM1_ESM.pdf]

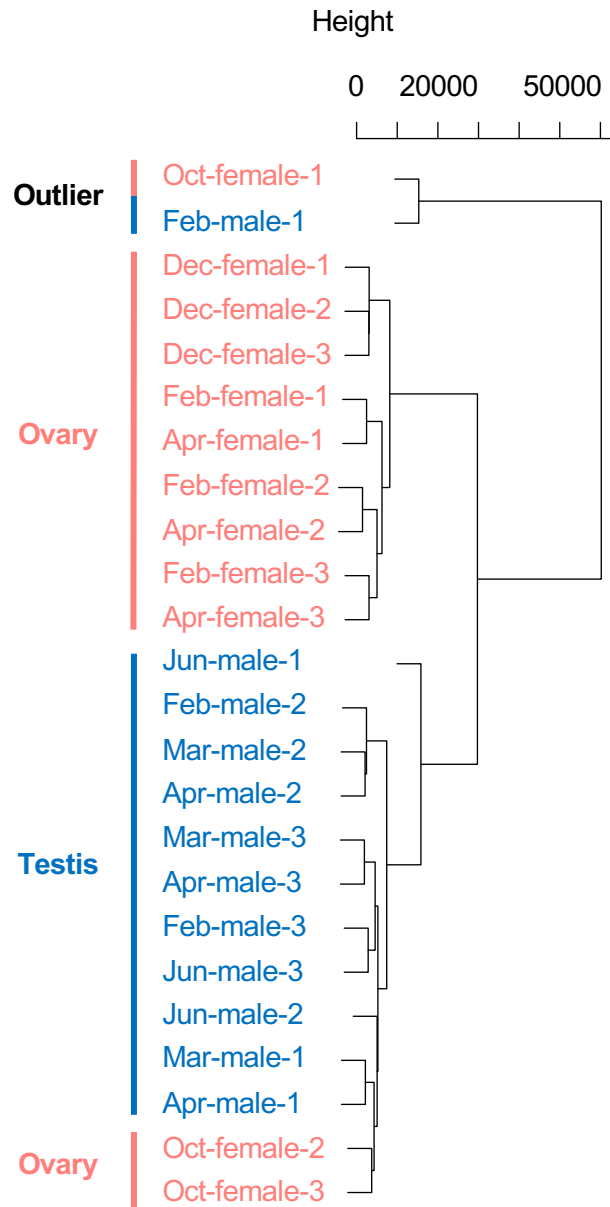

Supplement: Supplementary file 9 — Additional file 9 Hierarchical Clustering analysis of E. ancora gonadal samples used in this study. Twelve testis samples (3 colonies, 4 time points) and 12 ovary samples (3 colonies, 4 time points) were subjected to analysis. The cluster dendrogram showed that 2 samples (Oct-female-1 and Feb-male-1) are outliers, while others belong to a similar group. The 2 outliers were removed and the 22 remaining samples were used for gene expression analysis. [file 12864_2020_7113_MOESM9_ESM.pdf]

# Premature/mature ovaries

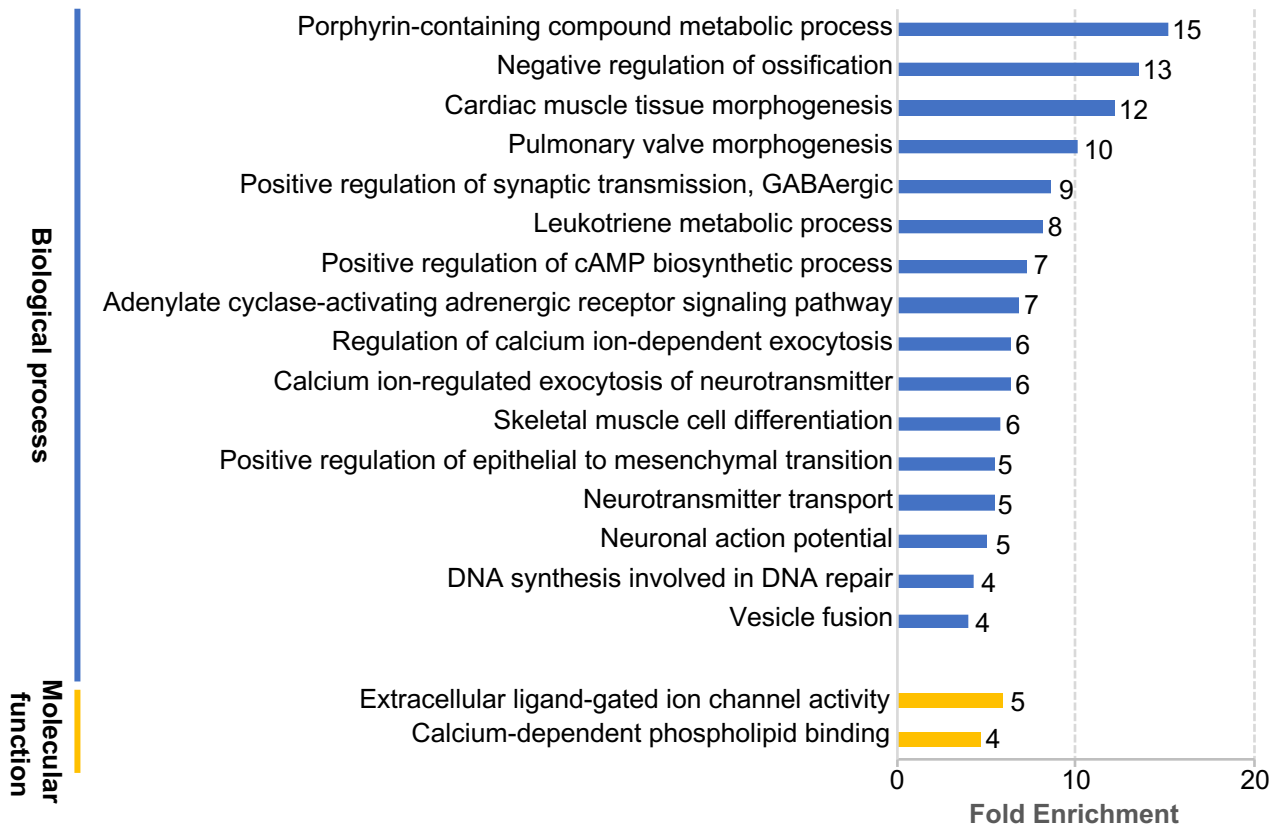

Supplement: Supplementary file 10 — Additional file 10 GO functional analysis of upregulated genes in premature/mature ovaries. Significantly (> 4-fold change, P < 0.05) enriched GO terms in biological processes (blue bar), and molecular function (yellow bar). The X-axis represents the magnitude of change. The Y-axis represents the GO functional category. [file 12864_2020_7113_MOESM10_ESM.pdf]

Mature testes

Biological process

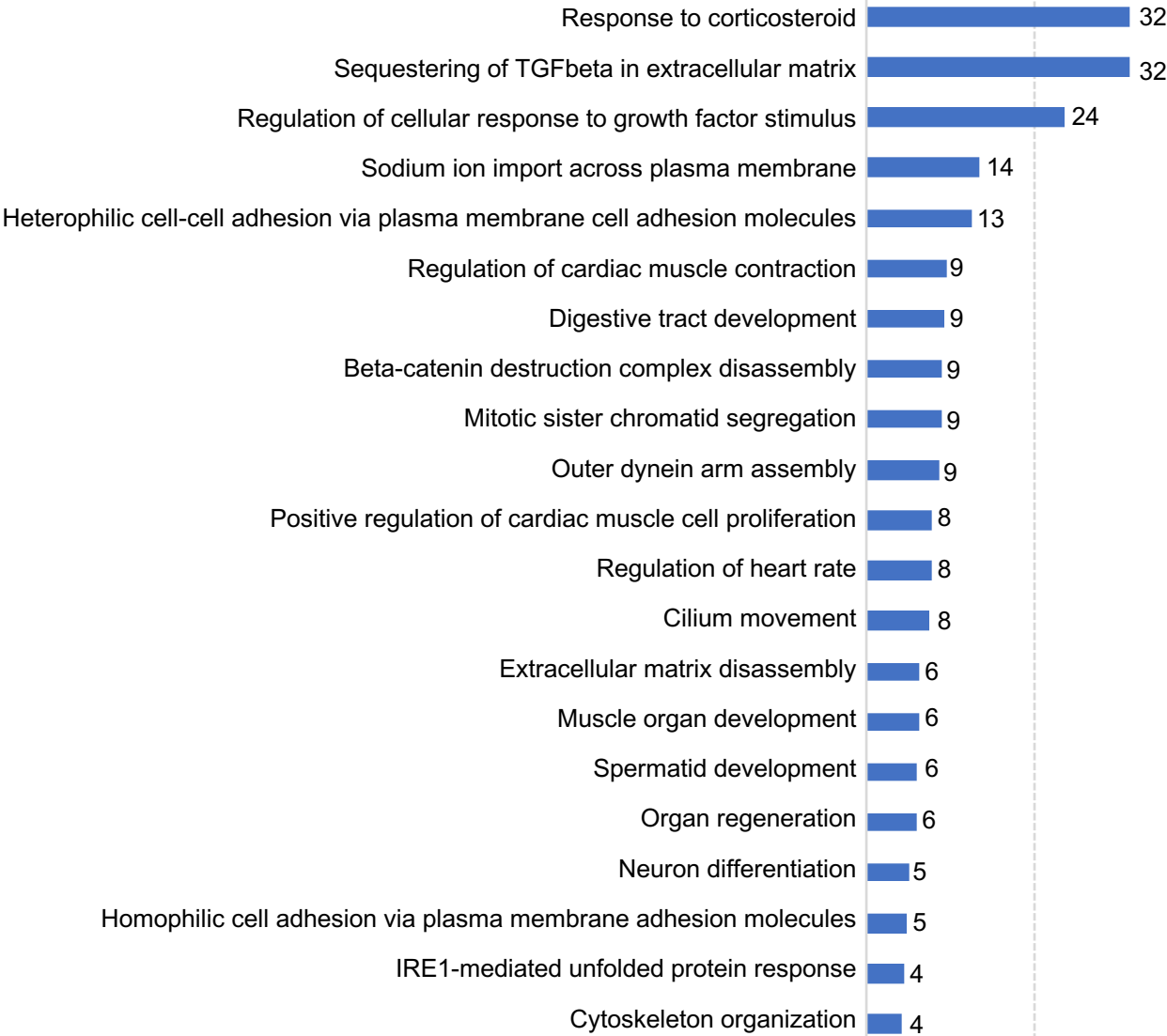

Cellular component

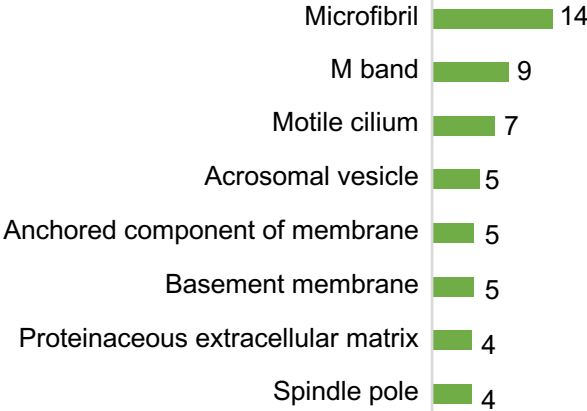

Molecular function

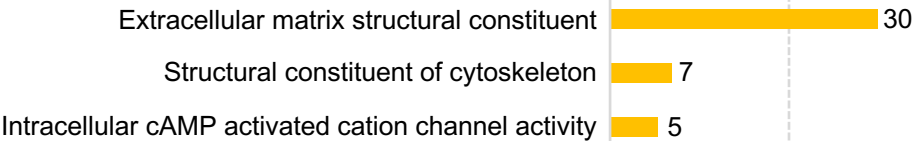

0 20 40  
Fold Enrichment

Supplement: Supplementary file 11 — Additional file 11. GO functional analysis of upregulated genes in mature testes. Significantly (> 4-fold change, P < 0.05) enriched GO terms in biological processes (blue bar), cellular component (green bar), and molecular function (yellow bar). The X-axis represents the magnitude of change. The Y-axis represents the GO functional category. [file 12864_2020_7113_MOESM11_ESM.pdf]
