# Supplementary material for: De novo transcriptome assembly from the gonads of a scleractinian coral, Euphyllia ancora: molecular mechanisms underlying scleractinian gametogenesis
Source: BMC Genomics. 2020 Oct 21;21:732. doi: 10.1186/s12864-020-07113-9 (PMC7579821; doi:10.1186/s12864-020-07113-9)
Supplement: Supplementary file 2 — Additional file 2. (Table) Summary of clean read data for 24 samples used in de novo assembly. [file 12864_2020_7113_MOESM2_ESM.pdf]

Summary of clean read data for 24 samples used in *de novo* assembly

Sample	Clean reads (million)	Clean bases (giga base pairs)	Q20 (%)	Q30 (%)	GC (%) content	Reads length (bp)
Oct-female-1	67.7425	10.1614	97.33	93.12	45.76	150
Oct-female-2	67.9405	10.1911	97.94	94.50	42.89	150
Oct-female-3	66.859	10.0289	97.39	93.34	43.13	150
Dec-female-1	67.843	10.1765	97.32	93.17	43.95	150
Dec-female-2	66.9528	10.0429	98.00	94.61	43.86	150
Dec-female-3	66.9538	10.0431	97.36	93.25	43.82	150
Feb-female-1	68.1661	10.2249	98.00	94.61	44.11	150
Feb-female-2	66.8133	10.022	98.01	94.61	44.13	150
Feb-female-3	66.7538	10.0131	97.37	93.24	44.15	150
Apr-female-1	66.736	10.0104	98.04	94.68	43.86	150
Apr-female-2	66.8641	10.0296	98.05	94.69	44.2	150
Apr-female-3	66.9086	10.0363	97.50	93.55	44.31	150
Feb-male-1	67.4389	10.1158	97.43	93.28	47.92	150
Feb-male-2	67.4123	10.1119	97.56	93.70	43.07	150
Feb-male-3	66.7983	10.0197	97.38	93.33	42.77	150
Mar-male-1	67.9211	10.1882	97.49	93.56	43.24	150
Mar-male-2	67.5062	10.1259	97.47	93.53	42.43	150
Mar-male-3	68.0462	10.2069	97.40	93.38	43.45	150
Apr-male-1	66.7666	10.015	97.45	93.47	43.17	150
Apr-male-2	67.0436	10.0565	97.57	93.73	43.2	150
Apr-male-3	67.2223	10.0833	97.55	93.69	43.34	150
Jun-male-1	67.2824	10.0924	97.49	93.53	43.75	150
Jun-male-2	67.564	10.1346	97.50	93.58	43.45	150
Jun-male-3	67.5078	10.1262	97.39	93.36	42.83	150

Q20 (%): The percentage of bases with Phred value >20

Q30 (%): The percentage of bases with Phred value >30
