# Supplementary material for: De novo transcriptome assembly from the gonads of a scleractinian coral, Euphyllia ancora: molecular mechanisms underlying scleractinian gametogenesis
Source: BMC Genomics. 2020 Oct 21;21:732. doi: 10.1186/s12864-020-07113-9 (PMC7579821; doi:10.1186/s12864-020-07113-9)
Supplement: Supplementary file 6 — Additional file 6 (Table) Reference databases used for identifying E. ancora-originated contigs from the E. ancora holobiont transcriptome assembly [file 12864_2020_7113_MOESM6_ESM.pdf]

Reference databases used for identifying *E. ancora*-originated contigs from the *E. ancora* holobiont transcriptome assembly

Category	Organism name	Project name	RefSeq category	RefSeq assembly accession	Reference
Scleractinian corals	*Acropora digitifera*	Adig_1.1	Genome	GCF_000222465.1	[31, 139]
	Pocillopora damicornis	ASM370409v1	Genome	GCF_003704095.1	[35, 140]
	Stylophora pistillata	*Stylophora pistillata* v1	Genome	GCF_002571385.1	[34]
	Orbicella faveolata	ofav_dov_v1	Genome	GCF_002042975.1	[141]
Symbiodiniaceae	*Symbiodinium* sp. A1	GAKY01	Transcriptome	GAKY01000001:GAKY01198297	[142]
	Symbiodinium sp. A2	GBGW01	Transcriptome	GBGW01000001:GBGW01057938	[143]
	Breviolum sp. B2	GBRZ01	Transcriptome	GBRZ01000001:GBRZ01087171	[143]
	Breviolum muscatinei	GFDR03	Transcriptome	GFDR03000001:GFDR03050575	[144]
	Uncultured *Cladocopium* sp.	IADM01	Transcriptome	IADM01000001:IADM01022773	[145]
	Uncultured *Durusdinium* sp.	IADN01	Transcriptome	IADN01000001:IADN01023426	[145]
