# Supplementary material for: De novo transcriptome assembly from the gonads of a scleractinian coral, Euphyllia ancora: molecular mechanisms underlying scleractinian gametogenesis
Source: BMC Genomics. 2020 Oct 21;21:732. doi: 10.1186/s12864-020-07113-9 (PMC7579821; doi:10.1186/s12864-020-07113-9)
Supplement: Supplementary file 7 — Additional file 7 (Table) Reproduction-related genes of E. ancora identified in our previous studies [file 12864_2020_7113_MOESM7_ESM.pdf]

Reproduction-related genes of *E. ancora* identified in our previous studies

Gene	Assembly ID	GenBank	Reference
Vasa2	Unigene20900_All	KT598028.1	[8]
Pl10	Unigene8229_All	JQ968406.1	[8]
Vitellogenin	CL4556.Contig6_All	KC777188.1	[12]
Egg protein	Unigene22577_All	KC777189.1	[12]
Piwi1	CL6184.Contig1_All	KJ820744.1	[9]
Piwi2	CL198.Contig64_All	KT598029.1	[9]
Euphy	Unigene22607_All	KR445656.1	[13]
Notch-like protein	Unigene4587_All	KR445658.1	[13]
Delta-like protein	Unigene121713_All	KR445657.1	[13]
Red fluorescent protein	CL12163.Contig1_All	KT452623.1	[15]
17beta hydroxysteroid dehydrogenase type 14	Unigene54416_All	KJ820745.1	[103]
DmrtE	Unigene124707_All	KP407877.1	[16]
Alpha-tubulin N-acetyltransferase	Unigene9360_All	KY947515.1	[21]
Alpha-tubulin	Unigene11886_All	KY908411.1	[21]
Green fluorescent protein	CL6640.Contig11_All	MG603733.1	[72]
Receptor guanylate cyclase A	CL4659.Contig4_All	MH894389.1	[22]
GLWamide preprohormone	Unigene184648_All	MG657359.1	[152]
Dmc1	Unigene30270_All	KT598032.1	Shikina et al. unpublished
Nanos 1	CL2131.Contig2_All	KT598030.1	Shikina et al. unpublished
Nanos 2	Unigene60208_All	KT598031.1	Shikina et al. unpublished
Vasa1	Unigene31450_All	JQ968407.1	Shikina et al. unpublished
Synaptonemal complex protein 2	CL8603.Contig1_All	KT598033.1	Shikina et al. unpublished
