# Supplementary material for: De novo transcriptome assembly from the gonads of a scleractinian coral, Euphyllia ancora: molecular mechanisms underlying scleractinian gametogenesis
Source: BMC Genomics. 2020 Oct 21;21:732. doi: 10.1186/s12864-020-07113-9 (PMC7579821; doi:10.1186/s12864-020-07113-9)
Supplement: Supplementary file 8 — Additional file 8 (Table) Evolutionarily conserved genes in metazoan reproduction identified in the E. ancora gonadal transcriptome assembly [file 12864_2020_7113_MOESM8_ESM.pdf]

Evolutionarily conserved genes in metazoan reproduction identified in the *E. ancora* gonadal transcriptome assembly

Category	Gene	Assembly ID	Accession	Reference
Germline development	*Germ cell-less (gcl)*	Unigene34697_All	M97933.1	[126]
	Mago nashi (mago)	Unigene124549_All	NM_057288	[127]
	Boule-like (boule)	CL3838.Contig1_All	HM222645.1	[128]
	Pumilio homolog 1 (pum1)	Unigene32425_All	NM_001159604.2	[129]
Meiotic process	*MutS protein homolog 4 (msh4)*	Unigene5502_All	NM_002440.4	[130-132]
	MutS protein homolog 5 (msh5)	Unigene44110_All	NM_172165.4	[130-132]
	DNA mismatch repair protein Mlh1	Unigene4354_All	AY090776.1	[132]
	Synaptonemal complex protein 1 (sycp1)	CL6324.Contig2_All	NM_012810.1	[133]
	Synaptonemal complex protein 3 (sycp3)	Unigene41119_All	NP_035647.2	[134]
	Double-strand-break repair protein rad21 (rad21)	CL3751.Contig1_All	NM_009009.4	[135, 136]
