# Supplementary material for: De novo transcriptome assembly from the gonads of a scleractinian coral, Euphyllia ancora: molecular mechanisms underlying scleractinian gametogenesis
Source: BMC Genomics. 2020 Oct 21;21:732. doi: 10.1186/s12864-020-07113-9 (PMC7579821; doi:10.1186/s12864-020-07113-9)
Supplement: Supplementary file 12 — Additional file 12. Schematic figures depicting domain structures of neurogenic locus notch homolog protein. (a) Neurogenic locus notch homolog proteins 1. (b) Neurogenic locus notch homolog proteins 2. (c) Neurogenic locus notch homolog proteins 3. [file 12864_2020_7113_MOESM12_ESM.pdf]

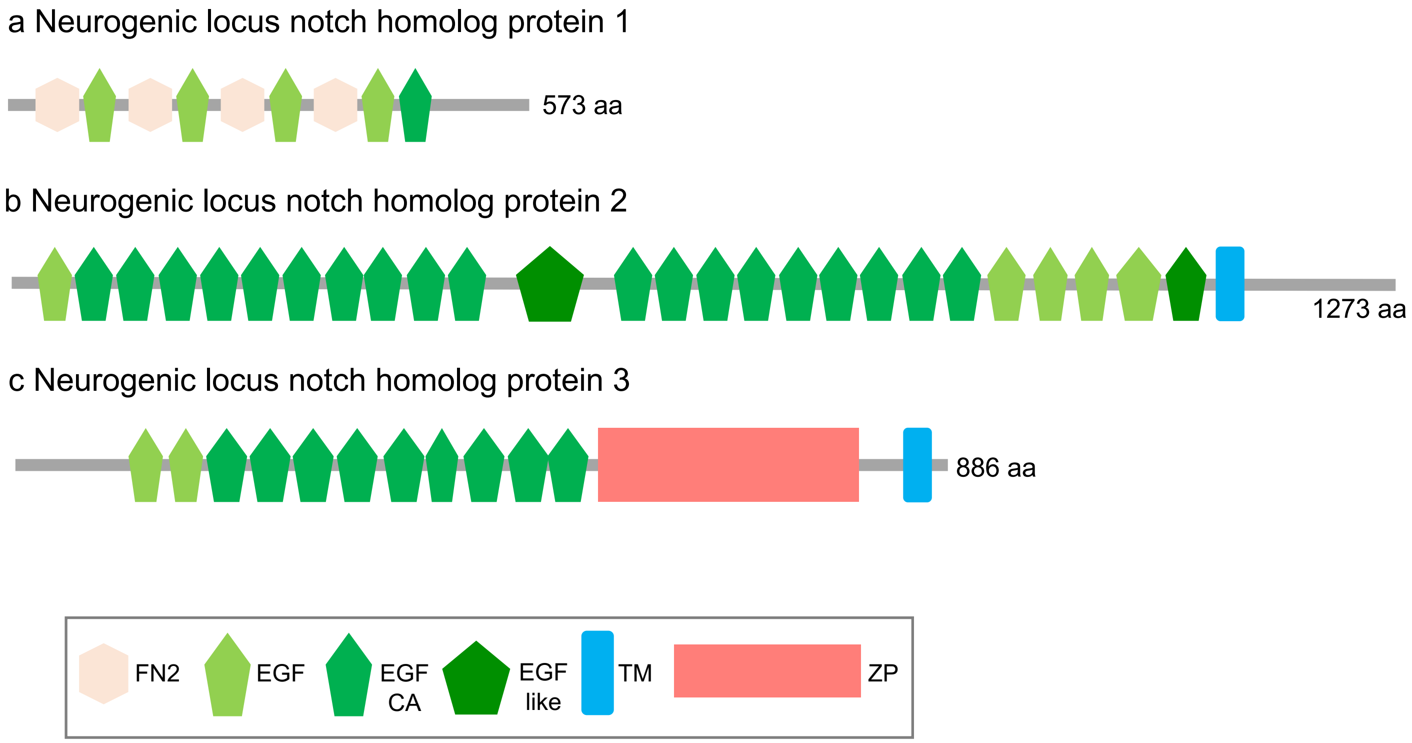

a Neurogenic locus notch homolog protein 1
573 aa
b Neurogenic locus notch homolog protein 2
1273 aa
c Neurogenic locus notch homolog protein 3
886 aa
FN2
EGF
EGF CA
EGF like
TM
ZP
